# Recovering Tomato Landraces to Simultaneously Improve Fruit Yield and Nutritional Quality Against Salt Stress

**DOI:** 10.3389/fpls.2018.01778

**Published:** 2018-11-30

**Authors:** Isabel L. Massaretto, Irene Albaladejo, Eduardo Purgatto, Francisco B. Flores, Félix Plasencia, Jose M. Egea-Fernández, Maria C. Bolarin, Isabel Egea

**Affiliations:** ^1^Department of Stress Biology and Plant Pathology, Centro de Edafología y Biología Aplicada del Segura, CEBAS-CSIC, Murcia, Spain; ^2^Department of Food Science and Experimental Nutrition, Faculty of Pharmaceutical Sciences, Food Research Center (FoRC-CEPID), University of São Paulo, São Paulo, Brazil; ^3^Department of Plant Biology, University of Murcia, Murcia, Spain

**Keywords:** *Solanum lycopersicum*, traditional varieties, salt tolerance, fruit quality, metabolites, carotenoids

## Abstract

Salt stress generally induces important negative effects on tomato (*Solanum lycopersicum*) productivity but it may also cause a positive effect improving fruit quality, one of the greatest challenges in nowadays agriculture. Because of the genetic erosion of this horticultural species, the recovery of locally adapted landraces could play a very important role in avoiding, at least partially, production losses and simultaneously improving fruit quality. Two tomato landraces endemic of the Spanish Southeast area, characterized by the harsh climatic conditions of the Mediterranean basin, have been selected: Negro Yeste (NY) characterized by its dark-red colored fruits and Verdal (V), which fruits did not achieve the characteristic red color at ripening. Here the agronomic, physiological, and metabolic responses of these landraces were compared with the reference tomato commercial cv. Moneymaker (MM), in plants grown without salt (control) and with salt stress (100 mM NaCl) for 70 days. The higher salt tolerance of both landraces was mainly reflected in the fruit number, as NY only reduced the fruit number in salt stress by 20% whereas in MM it was reduced till 43%, and in V the fruit number even showed an increase of 33% with salt stress. An important fruit quality parameter is soluble solids content, which increases induced by salinity were significantly higher in both landraces (60 and 78% in NY and V, respectively) compared with MM (34%). Although both landraces showed a similar response in relation to the high chlorophyll accumulation detected in their fruits, the fruit metabolic profiles were very different. Increased carotenoids levels were found in NY fruits, especially lycopene in ripe fruit, and this characteristic was observed in both control and salt stress. Contrarily, the carotenoid biosynthesis pathway was disrupted in V ripe fruits, but other metabolites, such as Ca^2+^, mannose, formate, and glutamate were accumulated. These results highlight the potential of tomato landraces to improve nutritional fruit quality and maintain fruit yield stability under salt stress.

## Introduction

Agriculture is probably facing its biggest challenge in human history due to world climate change, affecting global agricultural systems, especially in arid and semi-arid areas. In these areas salinization is a growing problem due to the frequent use of irrigation waters that contain salts. This risk will increase as population rises because cities and industry will pay for the best quality water, leaving the worst to agriculture. Therefore, development of crop plants tolerant to salt stress is vital to meet the growing food demand through sustainable agriculture when saline waters are used for irrigation. Nevertheless, a positive effect generally associated with salinity is the improvement of fruit quality (Cuartero et al., 2006). Given the predicted rise in world population fruits are expected to become the main source of secondary metabolites for millions of persons in near future. Therefore, the greatest challenge in next years will be to increase simultaneously crop production and fruit quality. The quality term is very wide and may refer to intrinsic and extrinsic characteristics, as well as to preharvest and postharvest periods (Kyriacou and Rouphael, 2018). The synthesis and accumulation of health-promoting metabolites, termed phytochemicals, depends mainly on the genetic material, although the agronomic practices and environmental factors also have an important influence on yield and quality characteristics of fruits and vegetables (Rouphael et al., 2012; Schreiner et al., 2013). Thus, salt and nutritional stresses have been used for the improvement of the nutritional quality of fruits (Colla et al., 2013; Fanciullino et al., 2014). However, to date progress has been largely limited to agronomic traits, whereas most of quality attributes, particularly those related to nutrition, have not been approached so deeply because of their complexity (Kyriacou and Rouphael, 2018; McQuinn et al., 2018).

Tomato (*Solanum lycopersicum*) is one of the most important horticultural crops worldwide (FAOSTAT, 2016). Aside from its socio-economic importance tomato has become a model species for fleshy fruits because of its agronomic and genetic features, and particularly as a rich plant source of carotenoids, vitamins, and minerals (Bergougnoux, 2014; Schwarz et al., 2014). Improving nutritional quality by enhancing the contents of bioactive compounds has become an important aspect for tomato fruit quality valorization and it has emerged as a challenge for growers who want to meet the ever-increasing demands of consumers in a highly competitive fresh market (Wu and Kubota, 2008). An important trait in breeding is improvement of individual carotenoid levels given their importance as precursors of volatiles associated with sensorial quality of tomato and as fundamental bioactive compounds in human health. Thus, β-carotene, the precursor of vitamin A, is essential for the health of eye while lycopene, the most abundant carotenoid in tomato, protects against chronic diseases and it diminishes the risk of cancer and cardiovascular diseases (Fraser and Bramley, 2004; Sharoni et al., 2012). The inability of humans to synthesize carotenoids *de novo* makes them dependent on plants as their primary source of dietary carotenoids. In a broad sense, the metabolome is what we assimilate from eating a tomato fruit that determines the nutritional value of this important crop. In this research work tomato is going to be used as a model crop since its fruit quality properties can be strongly modified by environmental conditions and, furthermore, it is the horticultural species supplementing the highest amount of metabolites in human diet given its so elevated consumption per capita (Cocaliadis et al., 2014; Liu et al., 2015).

One of the problems limiting the progress for developing tomatoes containing high levels of health-promoting compounds is genetic erosion (D'Esposito et al., 2017). Recently, Zhu et al. (2018) illustrated how breeding changed the tomato fruit metabolome. A serious consequence of biodiversity loss is the displacement of locally adapted landraces showing adaptation traits to harsh climactic conditions by genetically uniform hybrids and commercial cultivars (Frison et al., 2011). Plant breeding was long ago carried out by farmers who selected for specific adaptation traits leading to the generation of landraces. By contrast, modern plant breeding has emphasized adaptation to a wide spectrum of culture conditions, which has resulted in modern agriculture depending on a small number of cultivars for major crops. The main plant sources for food are more genetically vulnerable than ever before (Dwivedi et al., 2017). Because of their nearer genetic proximity to modern cultivars than to their wild relatives, landraces, or traditional varieties, may provide solutions for enhancing crop adaptation to abiotic stress as well as being new sources of healthy and nutritious food (Gascuel et al., 2017 and references therein). With this aim, two landraces adapted to adverse climatic conditions of the Spanish Southeast, with contrasted characteristics with respect to color and size of their fruits, were characterized at the agronomic, physiological, and metabolic levels in both control and salt stress conditions. The comparative response between the commercial tomato cv. Moneymaker and both landraces was analyzed in green and ripe fruits as well as in developed leaves close to the harvested fruit truss. We demonstrate that both landraces, exhibiting very different fruit metabolic profiles, are able to avoid, at least partially, the loss of fruit yield induced by salinity and at the same time to improve their fruit quality.

## Materials and methods

### Plant material and growth conditions

The tomato (*S. lycopersicum*) commercial cv. Moneymaker (MM) and the traditional varieties Negro Yeste (NY) and Verdal (V), collected in the semiarid area of Spanish Southeast, were used in this study. The morphological traits of the fruits (shape and size) were very different among them; MM, used as reference, is a round-shaped fruit type, whereas NY fruits are small size Pera type (cherry), and V ones are big size fruits and they have high locule number (Muchamiel type). The seeds of NY and V were supplied by the Agroecology Network of the Region of Murcia (RAERM).

Seeds were germinated in darkness, in a 2:1 (v/v) mixture of peat:perlite, at 28°C temperature and 90% of relative humidity (RH). After emergence, plants were grown in a controlled growth chamber with 16 h light/8 h darkness photoperiod, and 25°C and 50–60% of temperature and RH, respectively. A spring-summer culture was carried out in a glasshouse located at the campus of the University of Murcia (Espinardo, Region of Murcia, Spain), which offered us tight controlled culture conditions. The temperature was programmed to daily oscillate between 15 (night) and 28°C (day) and RH was maintained at 60%. At the 4th-leaf stage (30 days after sowing) 18 plants per variety were transplanted to plastic pots containing 17 L of coco peat (Projar Group,Valencia, Spain, 8 mm maximum particle size), using a drip irrigation system, with 3 L h^−1^ drippers (Supplementary Figure 1A). The fertigation solution (Hoagland solution, Hoagland and Arnon, 1950) was prepared in 2,000 L tanks with local irrigation water (EC = 0.9 dSm^−1^), and pH and EC was regularly monitored. The macronutrients salts used to prepare the nutrient solution were KNO_3_, Ca(NO_3_)_2_·H_2_O, NH_4_NO_3_, KH_2_PO_4_, MgSO_4_·7H_2_O; for micronutrients, Mn SO_4_·5H_2_O, H_3_BO_3_, Cu SO_4_·5H_2_O, (NH_4_)_6_Mo_7_O_24_·4H_2_O, and Zn SO_4_·7H_2_O were used, and Fe-DPTA (6%) for Fe.

At the 8th-leaf stage, salt treatment was applied to half of the plants for 70 days, by means of fertigation solution supplemented with 100 mM NaCl (PanReac AppliChem GmbH, Darmstadt, Germany), while the other half was irrigated without salt (control condition). Salt level was selected on the basis of previous studies (Campos et al., 2016; Egea et al., 2018) and its negative effect in the plants confirmed in preliminary experiments carried out under natural conditions (Egea et al., 2017). Lixiviate was collected with a frequency of 2 days once the salt treatment has started and EC and ionic composition was determined (Supplementary Figures 1A,B).

Differently of plants grown in our greenhouse under natural conditions, where a previous assay was carried out with three blocks, due to changing environmental conditions throughout the greenhouse (Egea et al., 2017), homogeneous environmental conditions were maintained in the whole culture surface of this glasshouse facility, which ensured us that differences were only due to two factors: genotype and salt treatment. A complete randomized design was used with nine plants per variety for each treatment (0 and 100 mM NaCl).

Ripe fruits from 2nd to 6th truss of each plant were collected, weighed and counted to estimate the two fruit yield components, fruit number and fruit weight. For ionomic and metabolic analyses, green fruits that had already reached their final size (green-mature stage, hereinafter named green fruits) and ripe fruits at commercial stage were harvested between the fourth and fifth trusses. In addition, samples of developed leaves located between these two trusses were also collected for these analyses. Except for color analysis, performed in fresh whole fruits, all samples (leaves, green fruits, and ripe fruits) were frozen and homogenized in liquid N_2_ and stored at −80°C until analysis. For each variety and treatment, three biological replicates were analyzed. In each replicate, leaves, green, or ripe fruits from three individual plants were pooled.

### Total soluble solids and color measurements

For the total soluble solid (TSS) content analysis, an aliquot of the tomato fruit samples were thawed and filtered through nylon membrane filter. Then the supernatant was collected and used to measure TSS using a refractometer with automatic temperature compensation (ATAGO PR-101 digital, Tokyo, Japan) and expressed as °Brix at 20°C. For each tomato subsample three technical replicates were measured. The color of fruit surface was measured by the Hunter Lab Color system (Hunter, 1975) using a chroma-meter (Minolta CR-400, Osaka, Japan).The color coordinates a^*^ (green-red) and b^*^ (yellow-blue) were determined and the a^*^/b^*^ ratio calculated.

### Total chlorophylls and carotenoids

In leaf measurements were determined by the method of Arnon (1949). Two hundred milligrams of freshly frozen leaves were homogenized in 20 mL 80% acetone. Homogenates were centrifuged at 3,000 × g for 10 min in a refrigerated centrifuge at 4°C. Absorbance of the supernatant was determined at 646, 663, and 470 nm, respectively. Chlorophylls and total carotenoids concentrations (μg/mL) were calculated according to the equations described by the method of Arnon (1949):

$$\begin{array}{lll} Chorophyll\;a\; & = & \;12.21\;A663-2.81\;A646\\ Chorophyll\;b\; & = & \;20.13\;A646-5.03\;A663\\ Carotenoids\; & = & \;1000\;A470-3.27\;Ca-104\;Cb \end{array}$$

In tomato fruits these pigments were determined by the method of Nagata and Yamashita (1992). Each freshly frozen sample (1.0 g) was homogenized with 20 mL of acetone:hexane (2:3), centrifuged at 3,000 × g for 10 min in a refrigerated centrifuge at 4°C. The optical density of the supernatant was spectrophotometrically determined at 663, 645, 505, and 453 nm. Calculations of chlorophylls and carotenoids (in mg/100 mL) were made according to the equations:

$$\begin{array}{l} Chorophyll\;a\;=\;0.999\;A663-0.0989\;A645\\ Chorophyll\;b\;=\;1.77\;A645-0.328\;A663\\ Carotenoids\;=\;0.216\;A663-1.22\;A645-0.304\;A505\\ \;+\;0.452\;A453 \end{array}$$

### Extraction and analysis of cations by ICP-OES

For the analysis of Na^+^, K^+^, and Ca^2+^contents dried lyophilized tissues were milled to powder, digested during 24 h in a concentrated HNO_3_: HClO_4_ (2:1 v/v) solution and analyzed by Inductively Coupled Plasma Optical Emission Spectrometry (ICP-OES) in a ICAP 6500 DUO/IRIS Intrepid II XLD equipment (Thermo Scientific, Waltham, MA, USA). Measurements were carried out at the Ionomics Service of CEBAS-CSIC (Murcia, Spain).

### Extraction and analysis of sugars and organic acids by ^1^H NMR

Aliquots of frozen samples were lyophilized over 48 h and then kept at −20°C in a closed recipient with Silicagel. The extraction protocol was based on Choi et al. (2004, 2006) with slight modifications. One mL of H_2_O:CH_3_OH 1:1 (v/v) solution was added to 50 mg of lyophilized material, then the mixture was vortexed for 1 min, sonicated for 1 min, and subsequently centrifuged (11,000 × g at 4°C for 20 min). The supernatant was collected in a 2 mL microtube and dried with a rotary vacuum evaporator. The dried extract was reconstituted in 800 μL of a D_2_O phosphate buffer (100 mM KH_2_PO_4_, pH = 6) containing 0.01% of TSP (0.58 mM trimethyl silyl propionic acid sodium salt) as internal standard and vortexed for 1 min. The mixture was centrifuged (16,100 × g at 4°C for 5 min) and 600 μL of the supernatant was transferred to an NMR tube for further analysis.

All ^1^H NMR spectra were recorded at 298 K on a Bruker AVIII HD 500 NMR spectrometer (500.13 MHz for ^1^H) equipped with a 5 mm CPP BBO cryogenic probe (Bruker Biospin, Germany). ^1^H spectra were referenced to TSP signal (δ = 0.00 ppm), whereas ^13^C spectra were referenced to CH-1 resonance of α-D-glucose (δ = 93.10 ppm). For each sample, 32 scans were recorded with the following parameters: 0.126 Hz/point, pulse width (PW) = 4.0 μs (30°) and relaxation delay (RD) = 1.0 s. FIDs were Fourier transformed with LB = 0.5 HZ, GB = 0, and PC = 1.0 and peak integral was used for quantitative analysis. The whole peak intensities in every 0.02 ppm in ^1^H NMR spectra in the range of δ 0.30–12.0 were used as variables. ^1^H NMR spectra were manually corrected for phase and baseline distortions using TOPSPIN (v3.2, Bruker Biospin). Peak-fitting on the resulting spectra was performed using a computer algorithm associated with Chenomx NMR Suite 8.1 software to generate concentrations of primary metabolites detected in plant material (Chenomx, Edmonton, AB, Canada). The region δ = 4.67–5.15 was discarded to eliminate the effects of imperfect water presaturation. The spectral areas of all buckets were normalized to the weight of extracts employed for measurements. The intensities of the selected ^1^H resonances due to hydro-alcoholic metabolites were measured with respect to the intensity of TSP signal used as internal standard with a concentration of 0.58 mM. Measurements were carried out at the Metabolomics Service of CEBAS-CSIC (Murcia, Spain).

### Extraction and analysis of carotenoids by UHPLC

Extraction of carotenoids was carried out using the method based on Sérino et al. (2009). One hundred microliters of 30% NaCl (w:v) solution was added to 200 mg (fruits) or 100 mg (leaves) of freshly frozen samples. The mixture was stirred for 1 min on a vortex agitator and then 200 μL of dichlorometane was added and stirred for 1 min. Subsequently, 500 μL of hexane:ether (1:1) was added and the mixture was stirred for 1 min and centrifuged (13,000 × g at 4°C for 5 min). The supernatant was collected in a 2 mL microtube; the procedure was repeated three times and the organic phases were pooled together. The remaining hexane phase was evaporated under N_2_ atmosphere. The dried carotenoid extract was reconstituted in 300 μL (fruits) or 900 μL (leaves) with the injection solvent [acetonitrile (ACN)/methanol (MeOH) 7:3, v/v]/acetone 6.7:3.3, v/v, for liquid chromatographic analysis. All sample solutions were filtered through Millex 0.2 μm nylon membrane syringe filters prior to their introduction into the UHPLC equipment (Millipore, Bedford, MA, USA).

UHPLC analyses were carried out using an Acquity I Class Ultra Performance LC system connected to a TUV detector measuring absorbance at 286 and 450 nm (Waters, Milford, MA, USA). UHPLC separations were performed on a reversed-phase column Acquity UPLC C18 BEH 130 Å, 1.7 μm, 2.1 × 100 mm (Waters), using the method described by Rivera et al. (2013). The mobile phase consisted of solvent A [ACN/MeOH 7:3 (v/v)] and solvent B (ultrapure water). The gradient used started by an isocratic 80% A: 20% B for 2 min and then performing a linear gradient up to 100% A over 1 min and maintained for 8.6 min, followed by a linear gradient down to 80% A: 20% B in 1 min and this equilibrium was held maintained for 2 min to stabilize the baseline. The column temperature was set at 32°C, the flow rate was 0.4 mL/min and the total runtime was 14.6 min including column equilibration.

Identification was carried out by comparison of retention time values and spectral properties of samples with those from authentic standards, purchased from Sigma Chemicals Co. (St. Louis, MO, USA) and CaroteNature (Lupsingen, Switzerland), and reference spectra. Standard stock solutions of major carotenoids present in leaves and fruits of tomato plants were prepared using HPLC-grade ethanol (neoxanthin, violaxanthin, and lutein) or hexane (phytoene, β-carotene, lycopene). Before use aliquots of each stock solution were diluted in their respective HPLC-grade solvent and each concentration was determined by UV-VIS absorption at their maximum absorbance wavelengths using the extinction coefficients (ε) described by Rivera and Canela (2012). Calibration was fulfilled by dose-response curves constructed from the standard solutions. Each concentration was determined by calculating the peak area and comparing it to the corresponding calibration curve. Measurements were carried out at the Metabolomics Service of CEBAS-CSIC (Murcia, Spain).

### Statistical analysis

Experimental data are presented as mean ± standard error (SE) of three biological replicates per variety and treatment. Statistical analysis was performed by two-way analysis of variance (ANOVA) and Tukey's test was applied to establish significant differences among mean values at *P* < 0.05, using the SPSS 24.0 software package. For multivariate analysis, PCA-biplot and heatmap were performed on these data matrixes and used to ascertain the overall variability among cultivars and treatments per tissue, i.e., leaves, green, and ripe fruits. Multivariate analysis was produced using the Metaboanalyst 4.0 server (Chong et al., 2018). Firstly raw data were normalized by median, processed using generalized log transformation (log 2) and then mean-centered and divided by the square root of deviation of each variable (Pareto scaling). The univariate analysis of fold change was also performed using the Metaboanalyst 4.0 server to evaluate significant differences among accumulated metabolites in fruits of landraces compared with MM.

## Results

### The tomato landraces showed enhanced salt tolerance together with improved fruit quality

**Figure 1:**
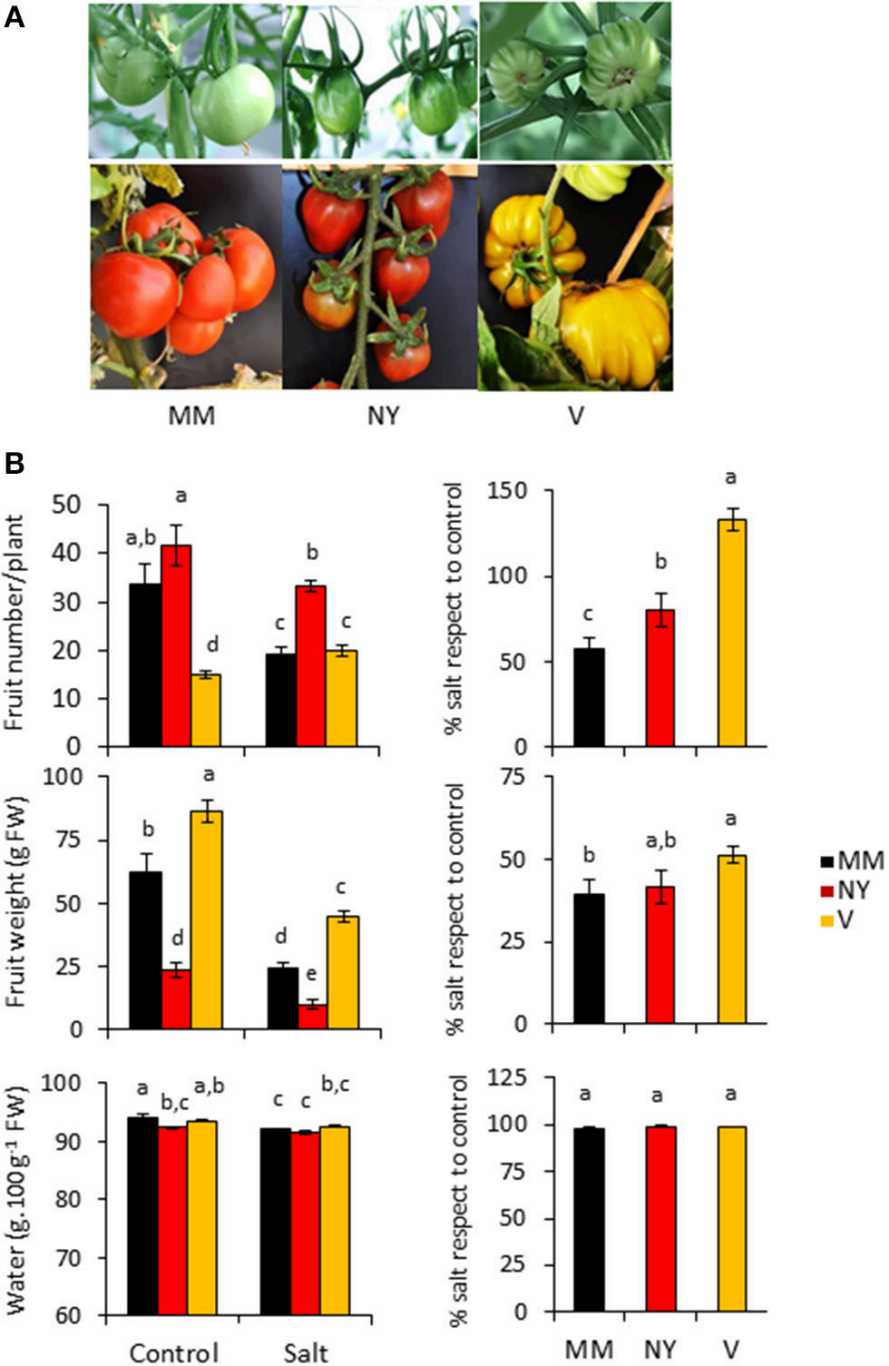
Fruit phenotype and fruit yield parameters of Negro Yeste (NY) and Verdal (V) landraces, and commercial cv. Moneymaker (MM). **(A)** Representative images of green and ripe fruits of each variety. **(B)** Fruit number, fruit weight, and fruit water content from plants grown without stress (control) and with salt stress (100 mM NaCl during 70 days). The relative values in salt stress with respect to control condition are presented at the right-hand side of the figure. Values are expressed as mean ± SE of nine plants per variety and treatment for fruit number and weight determinations, and of three biological replicates of 10 fruits each for fruit water content determination. Different letters indicate statistically significant differences (Tukey's test; *p* < 0.05).

The two tomato traditional varieties used in this study, Negro Yeste (NY) and Verdal (V), were initially selected because of their remarkable different fruit characteristics compared with the commercial cv. Moneymaker (MM). While NY fruits were smaller than those of MM and showed a darker red color, V fruits were of greater size and did not achieve the characteristic red color when ripened (Figure 1A). Although important differences were observed among the three varieties in both the number of fruits per plant as well as the average fruit weight, the two landraces were less affected by salinity than MM in the fruit number (Figure 1B, Supplementary Table S1). The relative values of fruit number in salt stress with respect to control condition were significantly lower in MM (57%), while in NY achieved a 80% and in V even became higher in salt treatment than in control condition (133%). Regarding fruit weight, however, only in V it increased significantly compared with MM. Therefore, the higher salt tolerance of both landraces is mainly reflected in the fruit number.

**Figure 2:**
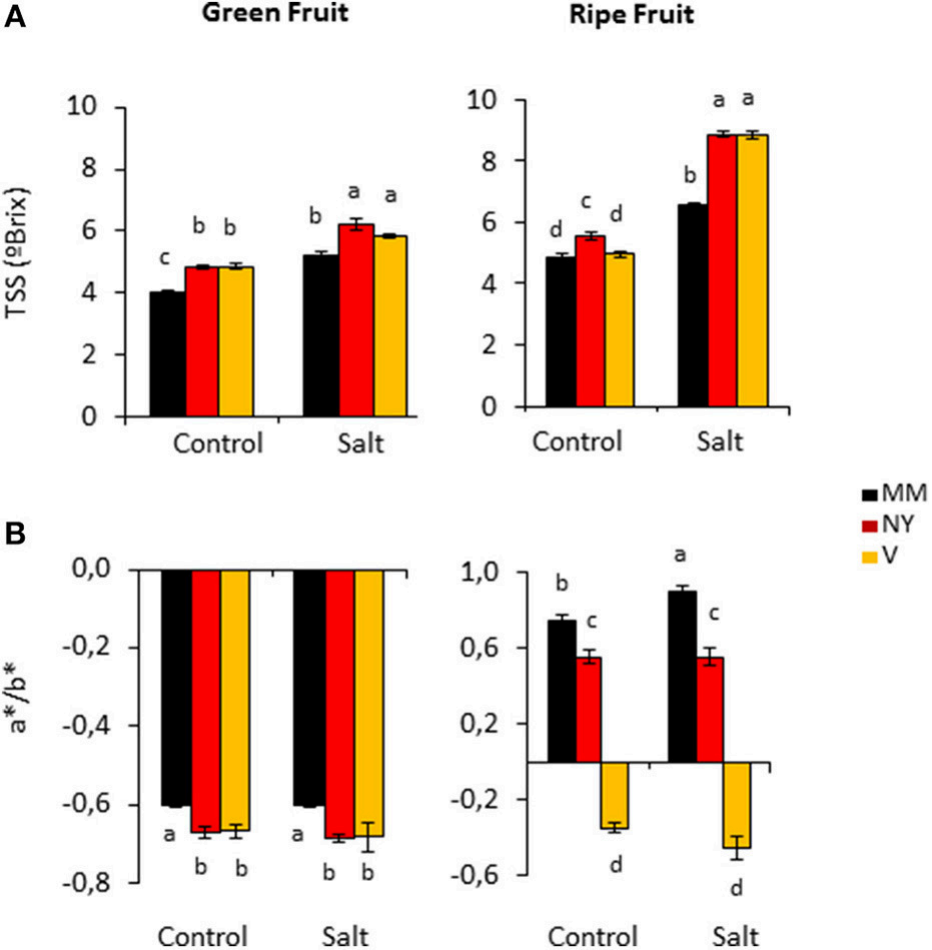
Quality characteristics of Negro Yeste (NY), Verdal (V), and Moneymaker (MM) green and ripe fruits from plants grown without stress (control) and with salt stress (100 mM NaCl during 70 days). **(A)** Total soluble solids (TSS) and **(B)** color evaluation expressed as a^*^/b^*^ ratio. Values are means ± SE of three biological replicates of ten fruits each. Different letters indicate statistically significant differences (Tukey's test; *p* < 0.05).

Total soluble solids (TSS) content is one of the most important quality parameters in tomato fruits; in fact the classification of tomato products, e.g., paste or puree, is done according to their TSS contents. Under control conditions, green fruits of both landraces displayed significantly higher TSS contents while in ripe fruits this was only observed in NY (Figure 2A, Supplementary Table S1). But the most interesting results were the significant TSS increases induced by salinity in ripe fruits of both landraces compared with MM (Figure 2A, Supplementary Table S1). Interestingly, this behavior was not due to an effect of solutes concentration because of dehydration since water contents of ripe fruits were similar in the three varieties under salinity condition (Figure 1B, Supplementary Table S1). Taken together, both landraces were able to partially avoid loss of fruit yield caused by salinity and to increase TSS content.

### Fruit color of the tomato traditional varieties reflected differences in pigments contents

Ripe fruits of the two landraces have visually different colors compared with MM, and consumers consider visual color as indicator of quality (Ringeisen et al., 2014). For fruit color analysis, a^*^/b^*^ coefficient was calculated from measured a^*^ and b^*^ color parameters (Figure 2B, Supplementary Table S1). a^*^/b^*^ coefficients were negative in green fruits of the three varieties under both control and salt stress conditions, indicating the presence of the characteristic green color prior to ripening. However, a^*^/b^*^ values were significant more negative in NY and V than in MM, which indicated a higher accumulation of green pigments in both landraces. The value of a^*^/b^*^ turned positive in ripe fruits of MM and NY, which reflected the characteristic change of color from green to red during fruit ripening. The increase of a^*^/b^*^ value was more pronounced in MM than in NY, pinpointing the fact that remaining presence of greenish tones together with red ones could be responsible of the visual red-brownish color observed in ripe NY fruits while MM fruits showed more pure red color (the highest a^*^/b^*^ value). Contrarily, ripe V fruits maintained negative values of a^*^/b^*^, which is in accordance to the characteristic orange-greenish color of this landrace.

**Figure 3:**
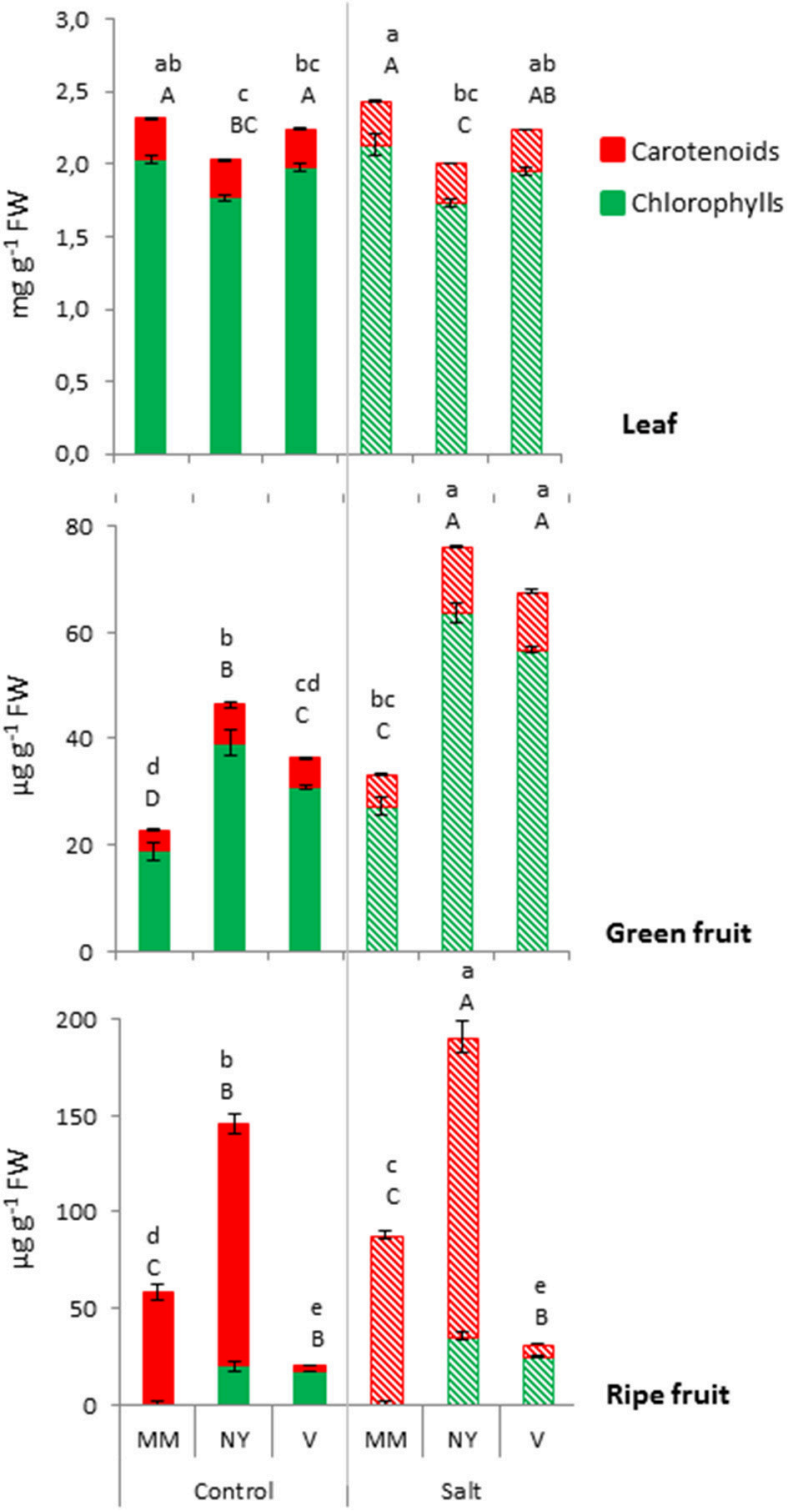
Total chlorophylls and carotenoids contents in leaf, green, and ripe fruits from Moneymaker (MM), Negro Yeste (NY), and Verdal (V) plants grown without stress (control) and with salt stress (100 mM NaCl during 70 days). Values are means ± SE of three biological replicates of ten fruits each. Different lowercase letters indicate significant differences in total carotenoids content while different capital case letters indicate significant differences in total chlorophylls content (Tukey's test; *p* < 0.05).

The color of tomato fruit during ripening is associated not only with carotenoids accumulation but also with chlorophylls degradation and, therefore, the differences in fruit color of the three varieties should be reflected in the pigment contents. Total contents of chlorophylls and carotenoids were measured in green and ripe fruits, and also in leaves, in order to know whether changes observed in fruits (sink organs) were related to changes in leaves (source organs) (Figure 3, Supplementary Table S1). While no significant differences in chlorophylls and carotenoids in MM and V leaves under control and salt conditions were detected, NY leaves showed the lowest values regarding both pigments. In green fruits both landraces showed a similar response as the chlorophylls and carotenoids contents significantly increased compared with MM, under control and, especially, under salt stress. Therefore, the high chlorophyll levels reflected the higher a^*^/b^*^ value in green fruits found in both landraces. As expected, the different color of ripe fruits of both landraces is associated to different changes in the pigments contents. The red-brownish color of NY ripe fruits was due to the very high degree of carotenoids accumulation together with persistent presence of chlorophylls. V fruits presented a completely different pattern concerning its carotenoids profile when ripening, as ripe fruits had much less carotenoids than MM in both control and salt stress, maintaining almost the same levels found in green fruits, which is reflected by the orange-greenish color when ripened. Finally, it is interesting to point out the much higher increase in carotenoids induced by salt treatment in NY ripe fruits compared with MM ones, which reflects a positive facet regarding nutritional quality of these fruits.

### Cations and metabolites contents in leaves

In order to know whether the higher degree of salt tolerance observed in NY and V compared with MM was related to their different ability for ion regulation, Na^+^, K^+^, and Ca^2+^ contents were analyzed in leaves from control and salt-treated plants for 70 days (Table 1, Supplementary Table S2). The K^+^/Na^+^, and Ca^2+^/Na^+^ ratios were also calculated, since high values of these parameters are related to salt tolerance in tomato. Under control condition V presented higher K^+^ content and NY higher Ca^2+^content than MM, but the most interesting result was the significantly lower Na^+^ accumulation induced by salinity in both landraces. V presented the highest K^+^/Na^+^, and Ca^2+^/Na^+^ ratios followed by NY, while MM showed the lowest values under salt stress.

**Table 1:** Contents of Na^+^, K^+^, and Ca^2+^ cations and K^+^/Na^+^ and Ca^2+^/Na^+^ ratio values in leaf, green, and ripe fruit from plants of cv. Moneymaker and traditional varieties Negro Yeste and Verdal grown under control and salt stress conditions (100 mM NaCl during 70 days).

**Cation**	**Moneymaker**		**Negro yeste**		**Verdal**	
	**Control**	**Salt**	**Control**	**Salt**	**Control**	**Salt**
**A) LEAF**						
Na^+^	227 ± 38^d^	1417 ± 58^a^	130 ± 10^d^	910 ± 13^b^	121 ± 2^d^	519 ± 8^c^
K^+^	2816 ± 42^b^	1150 ± 46^d^	2157 ± 160^c^	1504 ± 22^d^	3680 ± 63^a^	2821 ± 40^b^
Ca^2+^	3152 ± 52^b, c^	3153 ± 126^b, c^	4160 ± 300^a^	3615 ± 33^a, b^	2887 ± 41^c^	3238 ± 51^b, c^
K^+^/Na^+^	12.40 ± 0.02^c^	0.81 ± 0.01^f^	16.65 ± 0.06^b^	1.65 ± 0.01^e^	30.39 ± 0.07^a^	5.44 ± 0.01^d^
Ca^2+^/Na^+^	13.85 ± 0.01^c^	2.22 ± 0.01^f^	32.12 ± 0.20^a^	3.97 ± 0.02^e^	23.84 ± 0.02^b^	6.24 ± 0.01^d^
**B) GREEN FRUIT**						
Na^+^	44.78 ± 4.44^d^	267.42 ± 4.77^a^	32.64 ± 0.16^d^	181.57 ± 3.01^c^	30.29 ± 2.07^d^	207.65 ± 9.93^b^
K^+^	3058 ± 176^b^	4413 ± 53^a^	2979 ± 195^b^	4067 ± 338^a^	3078 ± 60^b^	4536 ± 112^a^
Ca^2+^	70.18 ± 10.21^a, b^	40.00 ± 4.50^c^	42.86 ± 6.76^c^	50.46 ± 8.12^b, c^	90.84 ± 9.07^a^	80.95 ± 4.13^a, b^
K^+^/Na^+^	69.20 ± 5.23^b^	16.52 ± 0.49^d^	91.30 ± 6.26^a^	22.36 ± 1.54^c^	102.78 ± 8.69^a^	21.92 ± 0.99^c^
Ca^2+^/Na^+^	1.57 ± 0.16^b^	0.15 ± 0.02^c^	1.31 ± 0.21^b^	0.28 ± 0.04^c^	3.06 ± 0.45^a^	0.39 ± 0.03^c^
**C) RIPE FRUIT**						
Na^+^	23.73 ± 2.16^c^	146.37 ± 8.94^a^	36.68 ± 1.04^c^	124.01 ± 7.51^a, b^	23.03 ± 1.87^c^	103.65 ± 3.88^b^
K^+^	2349 ± 340^a^	3161 ± 146^a^	3015 ± 88^a^	2510 ± 104^a^	2596 ± 167^a^	2537 ± 107^a^
Ca^2+^	30.72 ± 0.73^c, d^	35.78 ± 1.62^c^	55.50 ± 2.07^b^	23.77 ± 1.48^d^	76.76 ± 5.22^a^	52.93 ± 1.70^b^
K^+^/Na^+^	98.19 ± 6.52^a, b^	21.63 ± 0.32^c^	82.21 ± 0.08^b^	20.29 ± 0.52^c^	113.52 ± 6.95^a^	24.50 ± 0.83^c^
Ca^2+^/Na^+^	1.32 ± 0.16^b^	0.25 ± 0.02^c^	1.52 ± 0.10^b^	0.20 ± 0.01^c^	3.37 ± 0.31^a^	0.51 ± 0.03^c^

*Cation contents are presented as μg g^−1^ fresh weigh. Values are the mean of three biological replicates ± standard error. Different letters in the line indicate statistically significant differences (ANOVA, Tuckey's test; p < 0.05)*.

**Figure 4:**
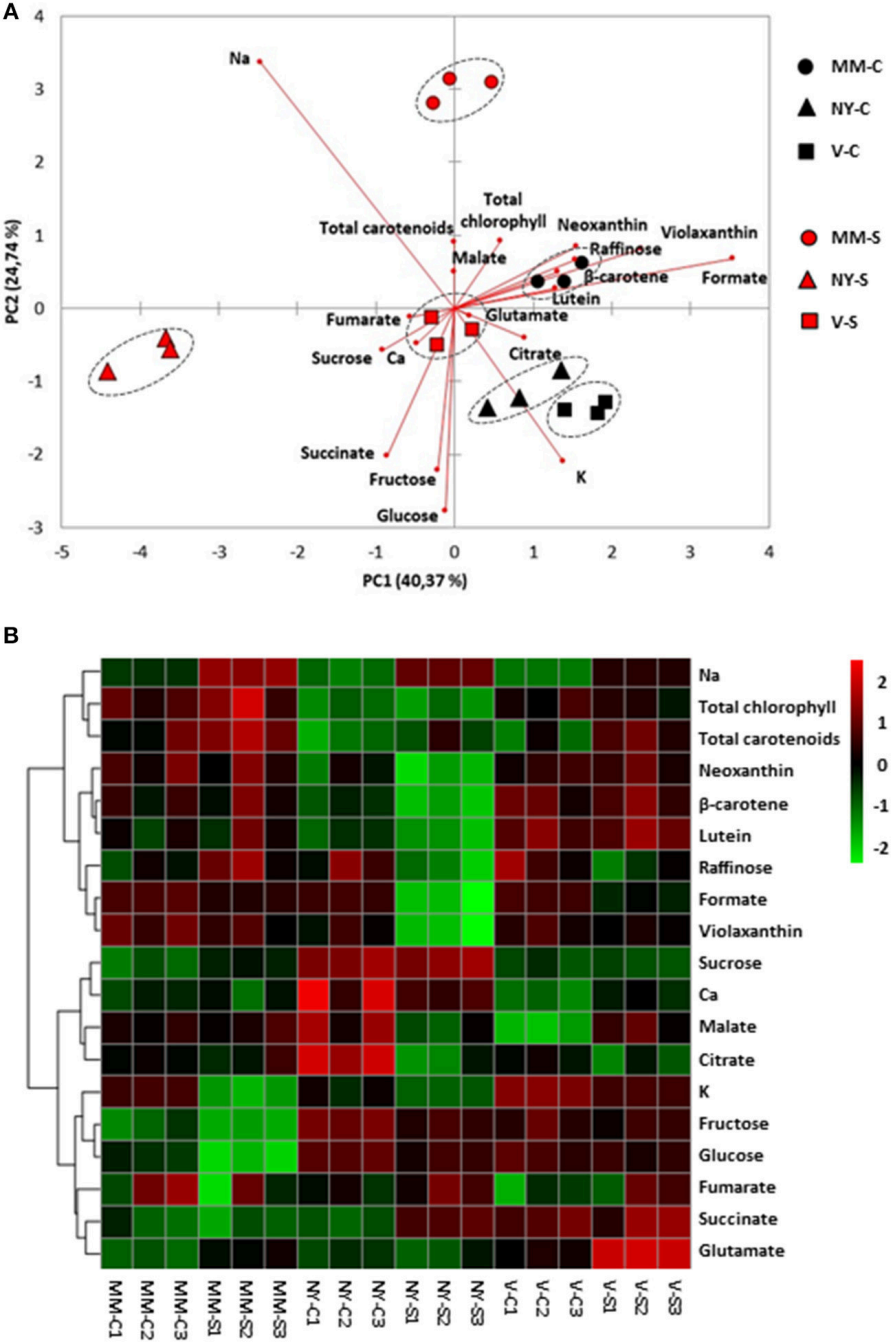
Relative metabolites and cations contents in leaves of Moneymaker (MM), Negro Yeste (NY), and Verdal (V) from plants grown under control and salt stress (100 mM NaCl during 70 days) conditions. **(A)** Non-supervised principal component analysis (PCA-biplot) and **(B)** heatmap analysis representing the major sources of variability. Color scale represents the variation in the relative concentration of compounds, from high (red) to low (green) contents.

Since metabolites may be translocated from leaves (source organs) to fruits (sink organs), the major primary metabolites, sugars, and organic acids, as well as the main carotenoids were analyzed in leaves (Table 2, Supplementary Table S2) and significant changes among varieties were observed in some of them. Sucrose accumulation was significantly higher in NY, both in control and salt conditions, while similar levels were found in MM and V. In order to analyze differences among varieties, a PCA-biplot on the whole dataset, including sugars, organic acids, pigments, and cations was performed (Figure 4A), and a heatmap analysis illustrating the variation in the relative concentration of each metabolite for the three varieties in control and salt-treated plants (Figure 4B). Under control condition, relative changes are similar enough among varieties since they are displayed very close in the PC1, which accounts for 40% of the total data variance. Under salt stress, NY was clearly separated from MM and V in PC1, mainly due to its lower contents of chlorophylls and carotenoids, and MM was separated from NY and V in PC2 (25% of the total variance), where the higher contents of glucose and fructose in both landraces were the main drivers for their separation from MM. Finally, it is interesting to point out the antagonism between Na^+^ and K^+^, as showed by their opposite arrows in the PCA-biplot.

**Table 2:** Metabolites contents in leaf, green, and ripe fruit from plants of cv. Moneymaker and traditional varieties Negro Yeste and Verdal grown under control and salt stress conditions (100 mM NaCl during 70 days).

**Metabolite**	**Moneymaker**		**Negro yeste**		**Verdal**	
	**Control**	**Salt**	**Control**	**Salt**	**Control**	**Salt**
**A) LEAF**						
Sucrose (mg g^−1^ fw)	0.44 ± 0.01^c^	0.49 ± 0.01^b^	0.64 ± 0.01^a^	0.64 ± 0.02^a^	0.46 ± 0.01^b, c^	0.46 ± 0.01^b, c^
Fructose (mg g^−1^ fw)	0.46 ± 0.03^c^	0.38 ± 0.01^c^	0.77 ± 0.01^a^	0.66 ± 0.02^b^	0.67 ± 0.04^a, b^	0.64 ± 0.02^b^
Glucose (mg g^−1^ fw)	0.29 ± 0.01^c^	0.17 ± 0.01^d^	0.46 ± 0.01^a^	0.40 ± 0.02^a, b^	0.43 ± 0.02^a, b^	0.40 ± 0.01^b^
Raffinose (mg g^−1^ fw)	0.27 ± 0.02^a, b, c^	0.40 ± 0.05^a^	0.37 ± 0.05^a, b^	0.18 ± 0.02^c^	0.39 ± 0.06^a^	0.23 ± 0.05^b, c^
Citrate	39.49 ± 0.56^b^	39.55 ± 3.01^b^	62.78 ± 2.88^a^	31.06 ± 3.11^b^	39.10 ± 1.07^b^	32.55 ± 2.57^b^
Succinate	2.94 ± 0.40^b^	2.46 ± 0.33^c^	2.79 ± 0.12^b^	6.62 ± 0.25^a, b^	7.01 ± 0.58^a, b^	7.74 ± 1.94^a^
Fumarate	1.25 ± 0.23^a^	0.92 ± 0.23^a^	0.96 ± 0.06^a^	1.22 ± 0.10^a^	0.77 ± 0.01^a^	1.10 ± 0.18^a^
Malate	68.56 ± 1.07^a, b^	69.58 ± 2.13^a, b^	78.64 ± 5.27^a^	60.82 ± 2.95^b, c^	50.36 ± 0.88^c^	71.36 ± 2.82^a, b^
Formate	8.55 ± 0.36^a^	5.89 ± 0.13^b^	7.15 ± 0.3^a, b^	1.7 ± 0.1^d^	7.6 ± 0.2^a^	3.5 ± 0.3^c^
Glutamate	77.5 ± 1.8^d^	109.9 ± 3.4^b, c^	89.6 ± 2.9^c, d^	85.6 ± 7.9^d^	116.6 ± 4.2^b^	238.4 ± 2.8^a^
Formate/succinate	3.03 ± 0.46^a^	2.48 ± 0.34^a^	2.57 ± 0.21^a^	0.11 ± 0.02^c^	1.10 ± 0.12^b^	0.51 ± 0.14^b, c^
Glutamate/succinate	27.29 ± 3.29^b^	46.30 ± 5.99^a^	32.23 ± 2.13^a, b^	12.88 ± 0.71^c^	16.80 ± 1.06^c^	36.83 ± 12.08^a, b^
β-carotene	25.99 ± 0.69^a^	26.92 ± 0.69^a^	22.01 ± 0.69^a, b^	17.25 ± 0.43^b^	28.52 ± 1.52^a^	28.98 ± 1.47^a^
Violaxanthin	11.56 ± 0.86^a^	9.45 ± 0.98^a, b^	8.50 ± 0.84^b^	3.26 ± 0.26^c^	9.54 ± 0.69^a, b^	8.22 ± 0.29^b^
Neoxanthin	8.80 ± 0.69^a^	8.49 ± 0.91^a^	6.60 ± 0.70^a, b^	4.52 ± 0.21^b^	8.10 ± 0.28^a^	8.54 ± 0.55^a^
Lutein	17.05 ± 0.65^b^	18.92 ± 0.65^a, b^	14.88 ± 0.65^b^	11.78 ± 0.45^c^	22.01 ± 1.23^a^	22.88 ± 1.32^a^
Chlorophyll a (mg g^−1^ fw)	1.49 ± 0.02^a, b^	1.54 ± 0.04^a^	1.33 ± 0.01^b^	1.30 ± 0.02^b^	1.46 ± 0.01^a, b^	1.38 ± 0.02^b, c^
Chlorophyll b (mg g^−1^ fw)	0.54 ± 0.01^a^	0.60 ± 0.03^a^	0.44 ± 0.01^b^	0.43 ± 0.01^b^	0.52 ± 0.02^a, b^	0.57 ± 0.01^a^
Chlorophyll:Carotenoid	7.23 ± 0.18^a^	7.17 ± 0.16^a^	6.91 ± 0.04^a, b^	6.42 ± 0.05^b^	7.49 ± 0.27^a^	6.79 ± 0.07^a, b^
**B) GREEN FRUIT**						
Sucrose (mg g^−1^ fw)	0.12 ± 0.01^c^	0.78 ± 0.04^b^	0.36 ± 0.04^b, c^	1.49 ± 0.11^a^	0.44 ± 0.05^b, c^	1.85 ± 0.20^a^
Fructose (mg g^−1^ fw)	8.5 ± 0.8^a^	8.0 ± 0.4^a^	8.9 ± 0.2^a^	8.9 ± 0.2^a^	7.5 ± 0.3^a^	7.0 ± 0.8^a^
Glucose (mg g^−1^ fw)	10.52 ± 1.28^a, b, c^	10.73 ± 0.49^a, b, c^	11.94 ± 0.40^a, b^	12.14 ± 0.16^a^	8.36 ± 0.26^b, c^	7.83 ± 1.18^c^
Mannose	11.74 ± 1.33^b^	15.69 ± 0.87^b^	14.79 ± 0.34^b^	17.07 ± 0.62^b^	12.37 ± 0.50^b^	32.67 ± 3.07^a^
UDP-glucose	34.66 ± 4.86^b^	35.66 ± 2.96^b^	40.48 ± 2.07^b^	40.14 ± 1.89^b^	46.78 ± 1.56^a, b^	54.83 ± 3.99^a^
Citrate (mg g^−1^ fw)	0.71 ± 0.06^c^	0.81 ± 0.05^b, c^	1.04 ± 0.05^a, b^	1.19 ± 0.08^a^	0.51 ± 0.05^d^	0.91 ± 0.01^b^
Succinate	41.27 ± 20.97^c^	138.32 ± 6.22^a^	89.07 ± 4.31^b^	126.59 ± 4.81^a^	37.66 ± 0.99^d^	45.28 ± 9.92^c^
Fumarate	5.44 ± 0.37^a^	3.47 ± 0.83^a, b^	5.92 ± 0.17^a^	4.43 ± 0.12^a, b^	3.26 ± 0.54^b^	3.78 ± 0.71^a, b^
Malate (mg g^−1^ fw)	1.70 ± 0.14^a, b^	1.90 ± 0.05^a^	1.69 ± 0.05^a, b^	1.40 ± 0.10^b, c^	0.98 ± 0.06^c, d^	0.96 ± 0.11^d^
Formate	1.8 ± 0.4^b^	2.3 ± 0.4^a, b^	1.3 ± 0.1^b^	1.4 ± 0.1^b^	2.2 ± 0.3^a, b^	2.8 ± 0.3^a^
Glutamate (mg g^−1^ fw)	0.17 ± 0.01^c^	0.29 ± 0.02^b^	0.19 ± 0.01^c^	0.33 ± 0.03^b^	0.24 ± 0.01^b^	0.57 ± 0.1^a^
Formate/succinate	0.06 ± 0.02^a^	0.02 ± 0.00^b^	0.02 ± 0.00^b^	0.01 ± 0.00^b^	0.06 ± 0.01^a^	0.07 ± 0.02^a^
Glutamate/succinate	6.25 ± 2.22^b^	2.07 ± 0.13^c^	2.14 ± 0.05^c^	2.59 ± 0.20^c^	6.39 ± 0.35^b^	12.64 ± 0.28^a^
Phytoene	2.02 ± 0.10^c^	2.69 ± 0.29^c^	4.96 ± 0.25^a, b^	6.18 ± 0.14^a^	2.71 ± 0.16^c^	4.63 ± 0.39^b^
β-carotene	1.21 ± 0.04^c^	1.22 ± 0.01^c^	1.39 ± 0.12^b^	2.01 ± 0.12^a^	1.65 ± 0.19^b^	1.78 ± 0.17^a, b^
Lutein	0.66 ± 0.02^c^	1.10 ± 0.08^b, c^	1.49 ± 0.15^b^	2.51 ± 0.28^a^	0.43 ± 0.04^d^	2.14 ± 0.17^a, b^
Chlorophyll a	14.14 ± 1.24^e^	20.69 ± 1.20^d^	28.75 ± 1.80^c^	46.99 ± 1.12^a^	22.49 ± 0.37^d^	41.31 ± 0.48^b^
Chlorophyll b	4.75 ± 0.48^d^	6.59 ± 0.43^c, d^	10.39 ± 0.69^b^	16.74 ± 0.69^a^	8.28 ± 0.02^b, c^	15.44 ± 0.14^a^
Chlorophyll:Carotenoid	4.80 ± 0.25^a^	4.57 ± 0.41^a^	5.45 ± 0.10^a^	5.11 ± 0.13^a^	5.59 ± 0.09^a^	5.17 ± 0.14^a^
**C) RIPE FRUIT**						
Sucrose (mg g^−1^ fw)	ND	0.10 ± 0.01^c^	0.05 ± 0.01^c^	0.30 ± 0.05^b^	0.16 ± 0.07^b, c^	0.75 ± 0.10^a^
Fructose (mg g^−1^ fw)	7.71 ± 0.54^c^	9.08 ± 0.23^b, c^	9.81 ± 0.37^a, b^	10.91 ± 0.32^a^	8.28 ± 0.28^b, c^	9.49 ± 0.21^a, b^
Glucose (mg g^−1^ fw)	10.67 ± 1.04^c, d^	13.37 ± 0.61^a, b^	11.65 ± 0.38^b, c^	14.92 ± 0.36^a^	8.95 ± 0.25^d^	12.17 ± 0.25^b, c^
Mannose	28.16 ± 2.92^b^	35.90 ± 6.14^b^	37.04 ± 3.64^b^	26.13 ± 3.93^b^	44.07 ± 2.37^a, b^	66.79 ± 9.02^a^
UDP-glucose	25.60 ± 3.62^a, b^	22.95 ± 0.65^a, b^	23.47 ± 0.75^a, b^	17.24 ± 1.62^b^	26.89 ± 4.77^a, b^	34.23 ± 6.23^a^
Citrate (mg g^−1^ fw)	0.76 ± 0.19^b^	1.27 ± 0.05^a^	1.25 ± 0.09^a^	0.99 ± 0.15^a, b^	0.73 ± 0.07^b^	0.46 ± 0.03^c^
Succinate	89.11 ± 8.59^b^	114.10 ± 9.07^a^	123.59 ± 9.22^a^	66.58 ± 7.53^b, c^	40.62 ± 8.19^c^	29.30 ± 5.78^c^
Fumarate	4.00 ± 0.38^a^	2.36 ± 0.84^a, b^	1.83 ± 0.43^a, b^	1.00 ± 0.182^b^	0.45 ± 0.04^c^	0.94 ± 0.26^b^
Malate (mg g^−1^ fw)	1.11 ± 0.20^b^	1.06 ± 0.12^b^	1.72 ± 0.07^a^	1.23 ± 0.10^b^	0.64 ± 0.02^c^	0.31 ± 0.04^d^
Formate	1.31 ± 0.05^c^	1.70 ± 0.18^c^	1.45 ± 0.05^c^	1.17 ± 0.02^c^	8.75 ± 1.62^b^	25.05 ± 1.18^a^
Glutamate (mg g^−1^ fw)	1.20 ± 0.15^b^	1.41 ± 0.05^a, b^	1.68 ± 0.14^a, b^	1.81 ± 0.14^a, b^	2.17 ± 0.30^a, b^	2.32 ± 0.32^a^
Formate/succinate	0.02 ± 0.00^c^	0.02 ± 0.00^c^	0.01 ± 0.00^c^	0.02 ± 0.00^c^	0.30 ± 0.20^b^	0.93 ± 0.20^a^
Glutamate/succinate	14.03 ± 1.30^d^	12.93 ± 1.84^d^	13.68 ± 0.31^d^	31.22 ± 7.92^c^	63.18 ± 16.83^b^	82.16 ± 11.95^a^
Phytoene	5.30 ± 0.36^b^	7.04 ± 0.18^a^	1.73 ± 0.11^d^	3.35 ± 0.12^c^	1.32 ± 0.17^d^	1.35 ± 0.02^d^
β-carotene	3.14 ± 0.10^b^	2.74 ± 0.19^b^	6.22 ± 0.18^a^	6.02 ± 0.15^a^	1.01 ± 0.12^c^	1.06 ± 0.07^c^
Lycopene	50.12 ± 4.20^d^	78.85 ± 2.24^c^	107.19 ± 4.41^b^	134.20 ± 8.15^a^	ND	ND
Lutein	0.25 ± 0.01^c^	0.36 ± 0.05^c^	1.36 ± 0.07^b^	2.12 ± 0.21^a^	0.35 ± 0.04^c^	0.65 ± 0.08^c^
Chlorophyll a	ND	ND	15.96 ± 2.06^b^	27.48 ± 2.16^a^	12.05 ± 0.56^b^	18.01 ± 2.00^b^
Chlorophyll b	ND	ND	4.21 ± 0.55^c^	8.13 ± 0.68^a^	5.11 ± 0.18^b, c^	6.76 ± 0.52^a, b^
Chlorophyll:Carotenoid	0.00 ± 0.00^d^	0.00 ± 0.00^d^	0.11 ± 0.02^c^	0.17 ± 0.02^c^	5.05 ± 0.21^a^	3.48 ± 0.36^b^

*Metabolites contents are presented as μg g^−1^ fresh weigh (fw) unless otherwise stated in the table. Values are the mean of three biological replicates ± standard error. Different letters in the line indicate statistically significant differences (ANOVA, Tuckey's test; p < 0.05). ND, not detected*.

### Cations and metabolites contents in green and ripe fruits

With regard to changes induced by salinity in cation contents in fruit, both landraces accumulated less Na^+^ than MM in green fruits, as was previously observed in leaves (Table 1, Supplementary Table S2). It is important to highlight the similar responses in leaves and green fruits of both landraces in spite of the much lower Na^+^ levels attained in fruits compared with leaves. Regarding other cations, it is also noteworthy the high Ca^2+^ content found in V, especially under salt stress. Moreover, the higher Ca^2+^ levels were also maintained in V ripe fruits and, consequently, high Ca^2+^ /Na^+^ ratio values were determined in fruits from this landrace.

**Figure 5:**
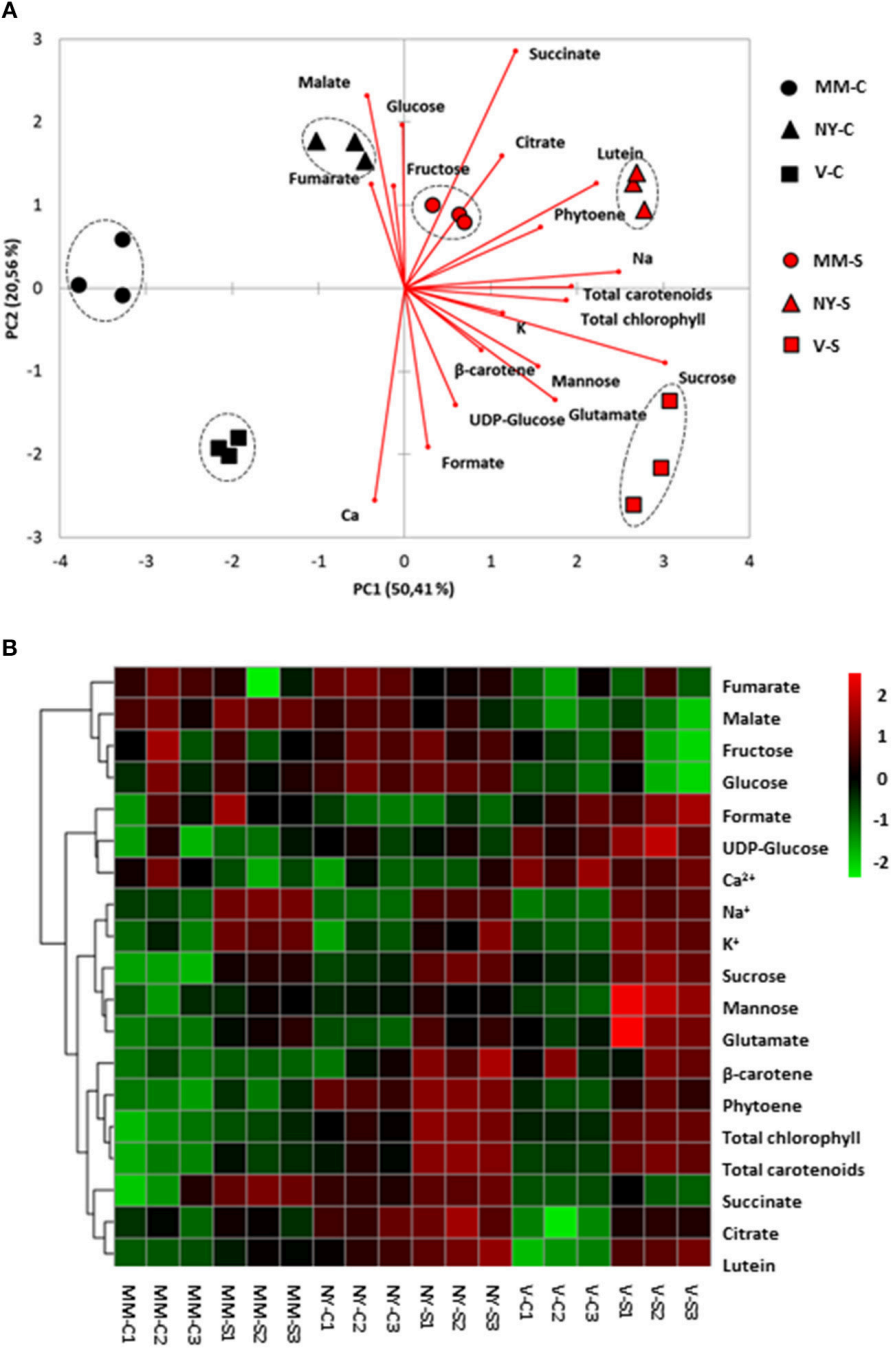
Relative metabolites and cations contents in green fruits of Moneymaker (MM), Negro Yeste (NY), and Verdal (V) from plants grown under control and salt stress(100 mM NaCl during 70 days) conditions. **(A)** Non-supervised principal component analysis (PCA-biplot) and **(B)** heatmap analysis representing of the major sources of variability. Color scale represents the variation in the relative concentration of compounds, from high (red) to low (green) contents.

In green fruits there were remarkable differences among varieties in the primary (sugars and organic acids) and secondary (carotenoids) metabolic profiles (Table 2, Supplementary Table S2). The PCA-biplot clearly separated the three varieties, where 50 and 20% of the total data variance was accounted for PC1 and PC2, respectively (Figure 5A). Na^+^ and metabolites such as sucrose, carotenoids, and chlorophylls greatly contributed to separate the samples by the PC1, while succinate, malate, formate, and Ca^2+^ were important compounds for the dispersion of the samples by the PC2. Interestingly, salt stress induced a remarkable effect on the metabolic composition of the three varieties by increasing the compounds contents but keeping their different metabolic signatures, as observed from heatmap analysis (Figure 5B). Green NY fruits presented higher contents of sucrose, citrate, and total chlorophyll in both conditions and succinate in control than MM. The biosynthetic pathway of carotenoids seems to be up-regulated in NY green tomatoes, since the phytoene content is two-fold higher, resulting in higher contents of lutein and β-carotene in this variety compared with MM (Table 2, Supplementary Table S2). V green fruits also produced sucrose, total chlorophyll, and carotenoids at higher levels compared with MM, but contrary to NY, the contents of organic acids citrate, malate, fumarate, and succinate were lower than in MM, particularly in control. The most interesting metabolic changes induced by salt stress in V green fruits were the increased levels of mannose and glutamate.

**Figure 6:**
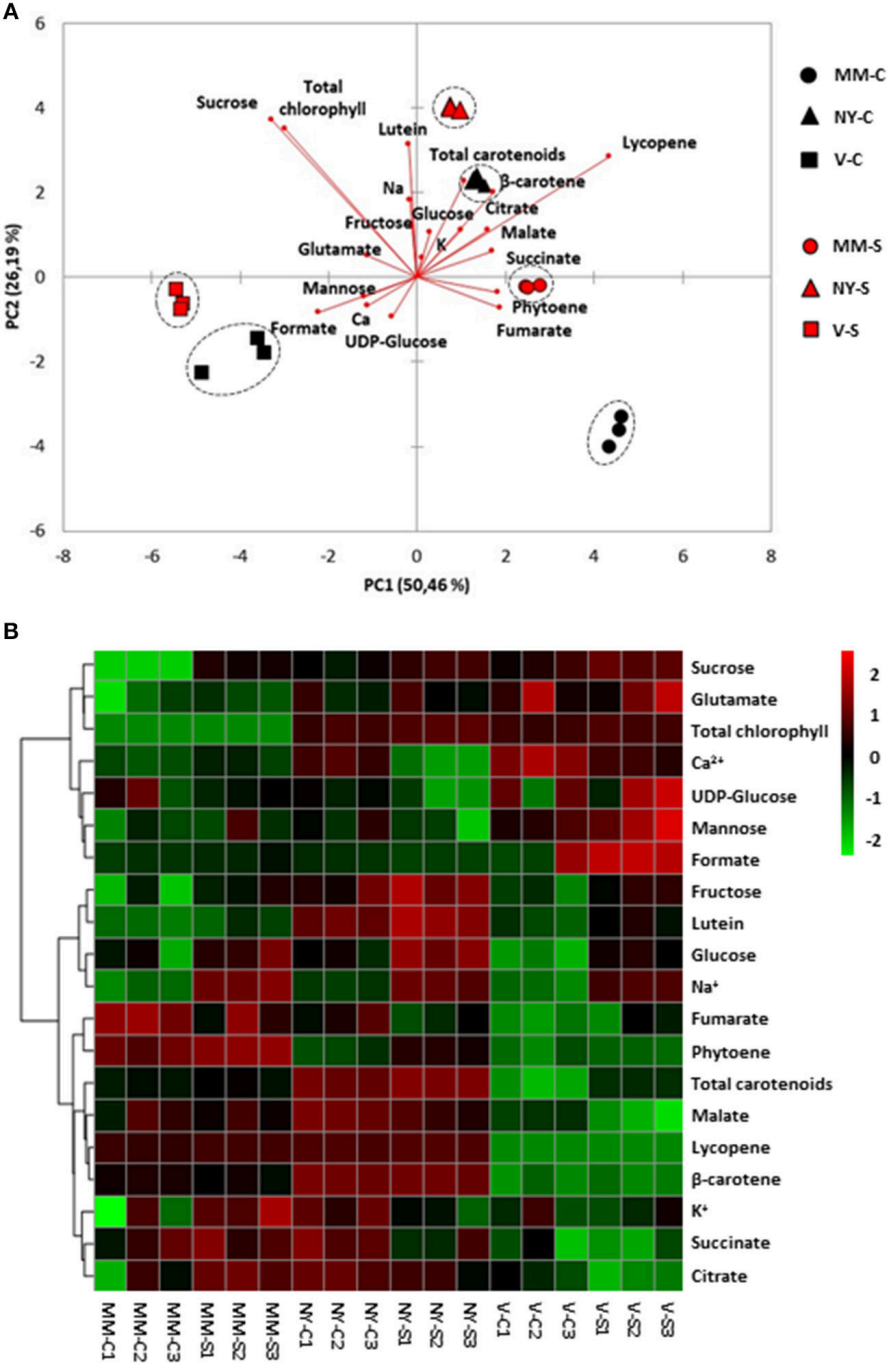
Relative metabolites and cations contents in ripe fruits of Moneymaker (MM), Negro Yeste (NY), and Verdal (V) from plants grown under control and salt stress (100 mM NaCl during 70 days) conditions. **(A)** Non-supervised principal component analysis (PCA-biplot) and **(B)** heatmap analysis representing the major sources of variability. Color scale represents the variation in the relative concentration of compounds, from high (red) to low (green) contents.

Ripe tomato fruit samples were displayed strongly separated on the PCA-biplot according to variety, which accounted for 50 and 26% of the total variance explained by PC1 and PC2, respectively (Figure 6A). It was observed that salt-treated samples remain close to their respective controls, indicating that the compounds profiles of MM, NY, and V seem to be more associated with genotype-specific variations than with those triggered by salt stress, in contrast with the salt response observed in green fruits. Basically, the higher contents of sucrose and chlorophylls in landraces were the main traits contributing to separate them from MM. Regarding carotenoids, lycopene, β-carotene, and lutein were the main metabolites increasing in NY compared with MM, especially under salt stress (Figure 6B, Table 2, Supplementary Table S2). However, the V ripe fruit pattern was very different, as only lutein content was similar to that found in MM, while β-carotene and phytoene were present in much lower concentrations and lycopene was not detected. But even more important differences were found when comparing V ripe fruits with MM ones; compounds from the TCA cycle as citrate, succinate and malate were sharply reduced while very high levels of formate were induced by salt stress, as observed also by the high value of the formate/succinate ratio attained in ripe fruits of this landrace (Table 2, Supplementary Table S2). It is also interesting to remark the high degree of accumulation of mannose detected in V ripe fruits, especially under salt stress. In sum, the metabolic composition of ripe fruits greatly differs between the two landraces and differences are also found when each landrace is compared with MM.

## Discussion

### The tomato traditional varieties improved cations homeostasis and increased sucrose and chlorophylls contents in fruits

In order to identify locally adapted traditional tomato varieties able to exhibit higher accumulation of compounds that positively influence nutritional quality and at the same time uphold fruit development under salt stress conditions, we have selected two landraces with very different fruit characteristics, showing lower reduction (NY) or even increased (V) fruit number under salinity compared with MM (Figure 1B). Although it is very difficult to elucidate which biological processes control plant and fruit growth and hence fruit yield (Sonnewald and Fernie, 2018), the higher salt tolerance of both landraces compared with MM was associated to increased K^+^/Na^+^ ratio values in leaves (Table 1), a physiological trait directly related with salt tolerance in tomato (García-Abellan et al., 2014). Interestingly, in spite of the much lower Na^+^ content achieved in fruits compared with leaves, the K^+^/ Na^+^ ratios in green fruits were maintained also at higher values in landraces than in MM. The Ca^2+^ changes observed may reflect how genotype and salinity affect transport via xylem from source leaves to sink fruits, as Ca^2+^ is the only ion transported to fruits mainly through the xylem (Gilliham et al., 2011). It could be observed in NY leaves a high Ca^2+^ level while in green fruit this is much reduced and differences between control and salt stress disappeared (Table 1). But the most significant changes detected regarding Ca^2+^ levels are the increases in V green and ripe fruits, which is of major interest from the point of view not only of plant stress response, as salt tolerance is associated to maintenance of Ca^2+^ homeostasis (Egea et al., 2018), but also from the nutritional quality perspective as this cation is very important in a healthy human diet. In addition, from a point of view of fruit quality, both landraces had a common characteristic: the similar TSS contents, which were significantly higher than MM, especially under salt stress. Moreover, this rising TSS content is not caused by a dehydration effect but it seems a *per se* accumulation of reducing sugars in the fruits (Figures 1B, 2A).

A key factor of fruit quality is the metabolic composition of this sink organ (Osorio et al., 2014), where an important portion of metabolites are imported from source leaves. However, we observed that the metabolic profiles were more specific of variety (genotype factor) in fruits than in leaves (Figure 4 compared with Figures 5, 6), which seems to indicate that final fruit quality is mainly the result of metabolic changes in fruits rather than in leaves. Moreover, it is in green fruits where the metabolite profiles of the three varieties were clearly modulated by salt stress. Thus, considering sucrose, the main carbon link between source and sink organs, it has been observed just a major constitutive level in NY leaves but no changes with salt stress in any of the three tomato varieties in the same organ. In fruits, however, sucrose levels were significantly higher in NY and V landraces compared with MM and, moreover, these levels increased with salt stress (Table 2). In this sense salinity tolerance of tomato has been related with higher sucrose transport from source organs to sink organs (Balibrea et al., 2000). But sucrose accumulation in fruits of both landraces may proceed partially because of enhanced exportation from source leaves and partially because of proper biosynthesis within the fruit, since fruit photosynthesis also contributes to sugar accumulation (Lytovchenko et al., 2011). Total chlorophylls were significantly higher in green fruits of NY and V compared with MM in control condition (108 and 63%, respectively), and this content even increased to a higher level under salt stress (134 and 108% in NY and V green fruits, respectively) (Figure 3). Taken together, the high levels of sucrose and chlorophylls found in fruits seem to be related with sustainable production under salt stress and with improved fruit quality independently of the environmental conditions.

### The improved fruit quality of negro yeste traditional tomato variety is associated to high carotenoids levels

**Figure 7:**
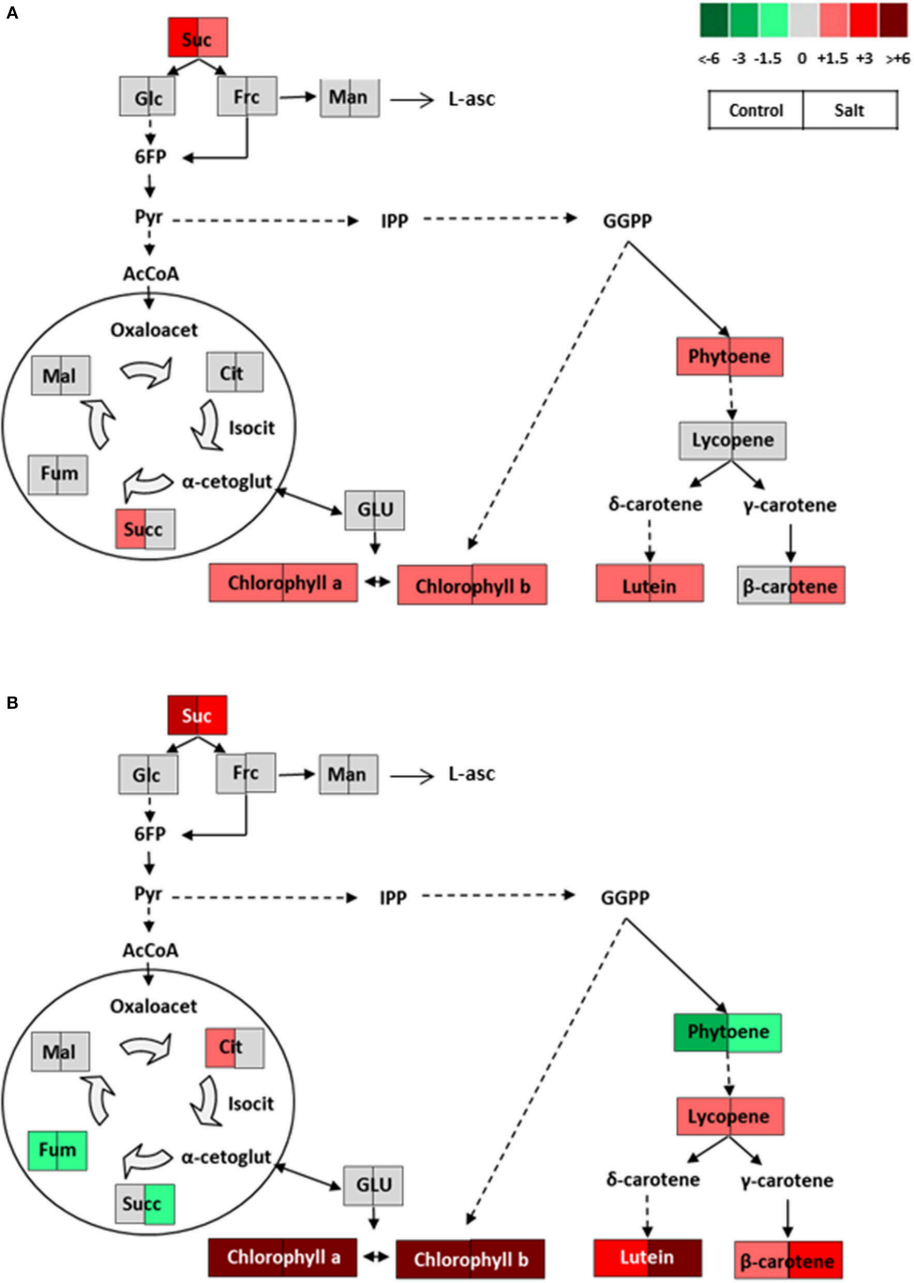
Schematic representations of the metabolic changes occurring in **(A)** green and **(B)** ripe fruits of NegroYeste from plants grown without stress (control) and with salt stress (100 mM NaCl during 70 days). Data were normalized to Moneymaker. Color scale is used to display the different amount of metabolite in terms of fold-change relative to the level in the appropriate control. Suc, sucrose; Glc, glucose; Frc, fructose; Man, mannose; L-asc, L-ascorbic acid; 6FP, fructose-6-phosphate; Pyr, pyruvate; IPP, isopentenyl diphosphate; GGPP, geranylgeranyl diphosphate; AcCoA, acetylCoA; Oxaloacet, oxaloacetate; Cit, citrate; Isocit, isocitrate; α-cetoglut, α-cetoglutarate; Succ, succinate; Fum, fumarate; Mal, malate; GLU, glutamate.

Changes in metabolites occurring in NY fruits compared with MM ones are represented in biochemical pathways for green and ripe fruits (Figure 7). Interestingly, the main changes are already observed in green fruits, as increased levels of sucrose, total chlorophylls, and phytoene, β-carotene and lutein carotenoids are found in control condition as well as in salt-treated plants. In ripe fruits, when lycopene accumulation from phytoene occurs in tomato fruits, the only difference observed with respect to green fruits is the phytoene reduction and lycopene rise in NY compared with MM, which reflects its higher capacity of biosynthesis of lycopene. Moreover, the contents of two carotenoids, β-carotene and lutein, were found at a higher level in fruits from salt-treated plants than from control ones. The idea that stress may affect the metabolism of carotenoids in tomato fruit makes sense because many carotenoids are powerful antioxidants and some of them are able to dissipate the excess of absorbed energy caused by the stressful condition in the xanthophyll cycle (Dall'Osto et al., 2013).

It is well-known that carotenoid biosynthesis and chlorophyll degradation pathways are closely related to color development in tomato fruit (Kang et al., 2017), and it is precisely the lack of chlorophyll degradation together with the high accumulation of lycopene what lead to fruits of dark red color in this NY variety. Other important questions to elucidate would be whether the high accumulation of carotenoids in NY fruits is a consequence of their high chlorophylls and sucrose levels. Regarding chlorophylls there are evidences that tomato fruits with more active chloroplasts at the green developmental stage can lead to ripe tomatoes with more active chromoplasts, producing higher amounts of carotenoids (Egea et al., 2010; Liu et al., 2015 and references therein). Moreover, carbohydrates may indirectly influence carotenoids through plastid development in fruits, pointing out that the sheer size of the biosynthetic machinery may be as important as the abundance or the activity of enzymes involved in the biosynthetic pathway of carotenoids (Fanciullino et al., 2014). Taken together the results found regarding carotenoids in relation with other metabolites in NY, this variety may serve as a model to advance in our knowledge of the key processes involved in carotenoid accumulation in tomato fruit. Moreover, it is interesting to point out that the high levels of carotenoids in NY fruits are found independently of the environmental conditions, that is to say, this landrace shows an improved fruit quality without stress and maintains and even increases its quality under a salt stress level relatively high for a sustainable production. In sum, NY could be useful for cultivation in a wide range of stress levels but especially moderate level, where fruit yield reduction would be minimum and, in case of salt stress, the salinity degree would be within a realistic agronomic scenario of arid and semi-arid cultivation areas where moderate saline waters are used in irrigation.

### The metabolism of ripe fruits of verdal traditional tomato variety, showing a disrupted carotenoids biosynthesis, is redirected toward formate accumulation

**Figure 8:**
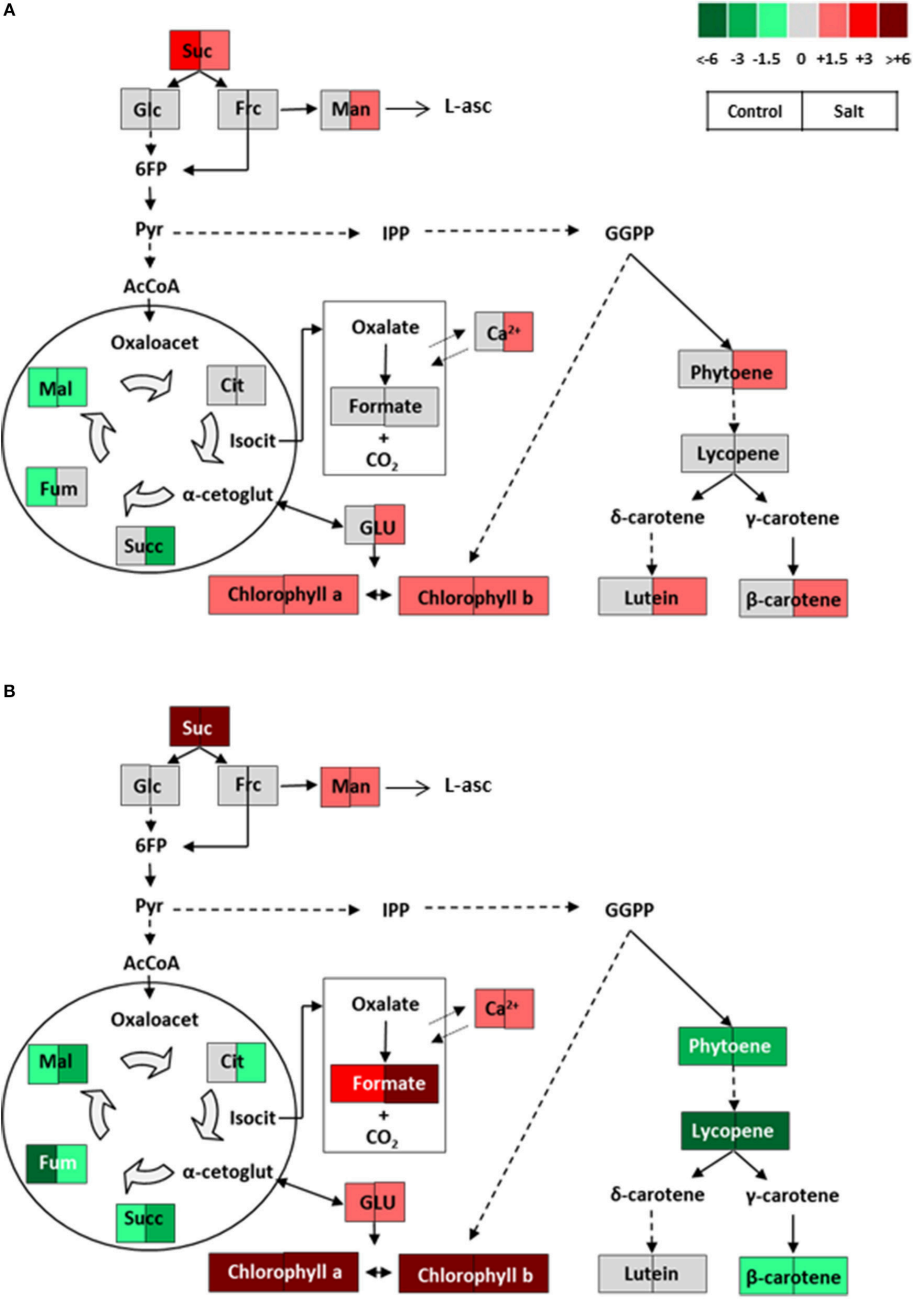
Schematic representations of the metabolic changes occurring **(A)** in green and **(B)** in ripe fruits of Verdal from plants grown without stress (control) and with salt stress (100 mM NaCl during 70 days). Data were normalized to Moneymaker. Color scale is used to display the different amount of metabolite in terms of fold-change relative to the level in the appropriate control. Suc, sucrose; Glc, glucose; Frc, fructose; Man, mannose; L-asc, L-ascorbic acid; 6FP, fructose-6-phosphate; Pyr, pyruvate; IPP, isopentenyl diphosphate; GGPP, geranylgeranyl diphosphate; AcCoA, acetylCoA; Oxaloacet, oxaloacetate; Cit, citrate; Isocit, isocitrate; α-cetoglut, α-cetoglutarate; Succ, succinate; Fum, fumarate; Mal, malate; GLU, glutamate.

In green fruits the schematic representation of metabolic changes found in V (Figure 8) is quite similar to that observed in NY (Figure 7), as levels of sucrose, chlorophylls, and carotenoids are higher in V compared with MM, although in case of the last group of metabolites the changes are limited to salt stress (Figure 8). Interestingly, the biggest differences between both landraces appear in the metabolic profiles of ripe fruits since, contrarily to NY, the V variety does not accumulate lycopene, an expected result according to the orange-greenish color of its ripe fruits, and levels of phytoene and β-carotene were reduced with respect to MM, being lutein the only carotenoid maintaining a similar level compared with the commercial cultivar. It is known that, in addition to carotenoids biosynthesis, several metabolic pathways are also derived from geranylgeranyl pyrophosphate (GGPP), like biosynthesis of chlorophylls and other key photosynthesis-related compounds (plastoquinones, phylloquinones, and tocopherols), as well as hormones (gibberellins, ABA, and strigolactones), and finally monoterpenes (Liu et al., 2015). In V fruits the disruption of carotenoids biosynthesis observed at ripe stage might induce the redirection of the metabolic flux toward other pathways. In addition to the high levels of chlorophylls, V fruits showed increased levels of glutamate and mannose in ripe fruits from plants grown in control and salt stress conditions (Figure 8). The glutamate rise observed in V fruits is interesting from a fruit quality point of view, since glutamate is a high-valued nutrient (Shinozaki and Ezura, 2016). Its high content could also be related to the higher accumulation of photosynthetic pigments, as this amino acid is the first precursor for the tetrapyrrole ring biosynthesis and an increase in its production may lead to an elevated flux toward chlorophyll accumulation (von Wettstein et al., 1995). Taking into account that the mannose metabolic pathway contributes to increase the ascorbate level in ripe tomato fruits (Badejo et al., 2012), the increased levels of mannose in V fruits might be related with biosynthesis of vitamin C (L-ascorbic acid).

In the “non-cyclic” partial TCA cycle, one branch produces citrate which can be transformed in isocitrate, 2-oxoglutarate or their derivatives (including glutamate), while the other branch produces malate or fumarate and even succinate (Igamberdiev and Eprintsev, 2016). According to the reduced levels of succinate, fumarate, and malate found in V fruits, the second branch was clearly reduced in fruits from this landrace, which could be associated with the increased sugars and total soluble solids observed in them (Centeno et al., 2011). However, V fruits could divert the metabolic flux to the other branch of the TCA, the one derived from isocitrate to render oxalate (Figure 8). One of the major pathways for efficient catabolism of oxalate is via its decarboxylation, as decarboxylases catabolize oxalate directly to formate, and CO_2_ (Chakraborty et al., 2013). We have observed remarkably high levels of formate in V ripe fruits and, moreover, this increase was higher in fruits from plants subjected to salt stress. Furthermore, the increased levels of formate and Ca^2+^ found in V fruits may be related, as Ca^2+^ is sequestered by oxalic acid, and if this organic acid is being catabolized at a higher degree, free Ca^2+^ is transported to the cytoplasm from the vacuole by tonoplast antiporters (Chakraborty et al., 2013). It is interesting to point out that most analytical studies about distribution of oxalates in plants have been focused on their possible Ca^2+^-sequestering activity and its influence in the human diet, as in its ionic form it performs critical functions in metabolism. Also Ca^2+^ deficiency is the most common nutritional problem affecting tomato fruit development (Nakata, 2003; Park et al., 2005). Finally, taking into account that tomato fruits accumulate considerable amounts of oxalate, a common anti-nutrient in the human diet, formate accumulation in ripe V fruits may be a very interesting trait in fruit quality breeding programs targeting tomato. Hitherto, the priorities in such breeding programs have been focused in rising primary and secondary metabolites, especially carotenoids, but in much less extension they have dealt with reduction of oxalic acid (Chakraborty et al., 2013).

In conclusion, both landraces show contrasting metabolic patterns to improve fruit quality, one (NY) increasing carotenoids and the other (V) redirecting the metabolic pathway toward other metabolites still unknown. Therefore, these varieties may be very interesting resources to be used in a near future to obtain new lines with improved fruit quality and enhanced fruit yield when grown under adverse environmental conditions.

## Author contributions

IM performed the experiments and data analysis, and contributed to the manuscript preparation. IA and FP helped in analysis of leaf and fruit materials. EP and FF critically revised the manuscript for important intellectual content. JE-F collected and selected the landraces. IE and MB conceived and designed the research and wrote the manuscript. All authors read and approved the manuscript.

### Conflict of interest statement

The authors declare that the research was conducted in the absence of any commercial or financial relationships that could be construed as a potential conflict of interest.
