# Supplementary material for: Recovering Tomato Landraces to Simultaneously Improve Fruit Yield and Nutritional Quality Against Salt Stress
Source: Front Plant Sci. 2018 Nov 30;9:1778. doi: 10.3389/fpls.2018.01778 (PMC6284034; doi:10.3389/fpls.2018.01778)
Supplement: Supplementary file 1 [file Table_1.pdf]

Supplementary Table S1. Average values of principal factors (genotype and condition) and *p*-values for these factors and their interaction for each parameter analyzed in figures 1B, 2 and 3.

Effect of the genotype and salt stress condition on fruit number, fruit weight and fruit water content (corresponding to results in fig. 1B).

		Fruit number / plant	Fruit weight	Fruit water content
Genotype (G)	Moneymaker	26.53^{b}	43.42^{b}	93.04^{a}
	Negro Yeste	37.50^{a}	16.70^{c}	91.94^{b}
	Verdal	17.52^{c}	65.53^{a}	93.04^{a}
	P-Value	***	***	**
Condition (C)	Control	30.16^{a}	57.41^{a}	93.28^{a}
	Salt Stress	24.21^{b}	26.28^{b}	92.06^{b}
	P-Value	***	***	***
Interaction	G x C	***	***	*

Effect of the genotype and salt stress condition on total soluble solids (TSS) and color evaluation expresses as a*/b* ratio (corresponding to results in fig. 2).

		GREEN FRUIT		RIPE FRUIT	
		TSS	a*/b*	TSS	a*/b*
Genotype (G)	Moneymaker	4.64^{b}	-0.60^{a}	5.70^{c}	0.82^{a}
	Negro Yeste	5.54^{a}	-0.68^{b}	7.18^{a}	0.55^{b}
	Verdal	5.34^{a}	-0.67^{b}	6.90^{b}	-0.40^{c}
	P-Value	***	***	***	***
Condition (C)	Control	4.58^{b}	-0.64	5.12^{b}	0.32
	Salt Stress	5.77^{a}	-0.66	8.07^{a}	0.34
	P-Value	***	n.s.	***	n.s.
Interaction	G x C	*	*	***	*

Effect of the genotype and salt stress condition on total chlorophylls and carotenoids contents in leaf, green and ripe fruits (corresponding to results in fig. 3).

		LEAF		GREEN FRUIT		RIPE FRUIT	
		Chlorophylls (mg g^{-1} fw)	Carotenoids (mg g^{-1} fw)	Chlorophylls	Carotenoids	Chlorophylls	Carotenoids
Genotype (G)	Moneymaker	2.08^{a}	0.29^{a}	23.08^{c}	4.97^{c}	n.d.^{c}	73.25^{b}
	Negro Yeste	1.75^{c}	0.26^{b}	51.44^{a}	9.83^{a}	27.89^{a}	140.05^{a}
	Verdal	1.96^{b}	0.28^{a,b}	43.76^{b}	8.25^{b}	20.96^{b}	5.27^{c}
	P-Value	***	***	***	***	***	***
Condition (C)	Control	1.93	0.27^{b}	29.60^{b}	5.54^{b}	12.44^{b}	62.37^{b}
	Salt Stress	1.94	0.28^{a}	49.26^{a}	9.83^{a}	20.12^{a}	83.35^{a}
	P-Value	n.s.	**	***	***	***	***
Interaction	G x C	*	*	***	***	**	*

Pigments contents are presented as μg g^{-1} fresh weigh (fw) unless otherwise stated in the table. Different letters in each principal factor indicate statistically significant differences at $P < 0.05$ (Tukey's test). *, ** and *** indicates significant differences at $P \leq 0.05$, $P \leq 0.01$ and $P \leq 0.001$ respectively for each main factor and the interaction. n.s. = no significance.
