# Supplementary material for: Recovering Tomato Landraces to Simultaneously Improve Fruit Yield and Nutritional Quality Against Salt Stress
Source: Front Plant Sci. 2018 Nov 30;9:1778. doi: 10.3389/fpls.2018.01778 (PMC6284034; doi:10.3389/fpls.2018.01778)
Supplement: Supplementary file 2 [file Table_2.pdf]

Supplementary Table S2. Average values of principal factors (genotype and condition) and *p*-values for these factors and their interaction for each parameter analyzed in tables 1 and 2.

Effect of the genotype and salt stress condition on the contents of Na^+, K^+ and Ca^{2+} cations and K^+/Na^+ and Ca^{2+}/Na^+ ratio values in leaf, green and ripe fruit (corresponding to results in table 1).

**LEAF**		Na^+	K^+	Ca^{2+}	K^+/Na^+	Ca^{2+}/Na^+
Genotype (G)	Moneymaker	822[a]	1983[b]	3153[b]	6.60[c]	8.05[c]
	Negro Yeste	520[b]	1831[b]	3888[a]	9.15[b]	18.04[a]
	Verdal	319[c]	3251[a]	3063[b]	17.92[a]	15.04[b]
	P-Value	***	***	***	***	***
Condition (C)	Control	159[b]	2884[a]	3400	19.81[a]	23.28[a]
	Salt Stress	949[a]	1825[b]	3336	2.63[b]	4.15[b]
	P-Value	***	***	n.s.	***	***
Interaction	G x C	***	***	*	***	***
**GREEN FRUIT**						
Genotype (G)	Moneymaker	156.10[a]	3736	55.09[b]	42.86[b]	0.86[b]
	Negro Yeste	107.11[b]	3523	46.66[b]	56.83[a]	0.80[b]
	Verdal	118.97[b]	3807	85.90[a]	62.35[a]	1.72[a]
	P-Value	***	n.s.	***	**	***
Condition (C)	Control	35.90[b]	3038[b]	67.96	87.76[a]	1.98[a]
	Salt Stress	218.88[a]	4339[a]	57.14	20.27[b]	0.27[b]
	P-Value	***	***	n.s.	***	***
Interaction	G x C	***	*	*	*	**
**RIPE FRUIT**						
Genotype (G)	Moneymaker	85.05[a]	2755	33.25[b]	59.91[a,b]	0.786[b]
	Negro Yeste	80.35[a]	2763	39.64[b]	51.25[b]	0.855[b]
	Verdal	63.34[b]	2567	64.85[a]	69.01[a]	1.943[a]
	P-Value	**	n.s.	***	**	***
Condition (C)	Control	27.81[b]	2653	54.33[a]	97.97[a]	2.07[a]
	Salt Stress	124.68[a]	2736	37.50[b]	22.14[b]	0.32[b]
	P-Value	***	n.s.	***	***	***
Interaction	G x C	**	n.s.	***	*	***

Cation contents are presented as $\mu g\ g^{-1}$ fresh weigh. Different letters in each principal factor indicate statistically significant differences at $P < 0.05$ (Tukey's test). *, ** and *** indicates significant differences at $P \leq 0.05$, $P \leq 0.01$ and $P \leq 0.001$ respectively for each principal factor and the interaction. n.s. = no significance.

Effect of the genotype and salt stress condition on metabolites contents in leaf of commercial cv. Moneymaker and traditional varieties Negro Yeste and Verdal (corresponding to results in table 2).

A		Sucrose (mg g^{-1} fw)	Fructose (mg g^{-1} fw)	Glucose (mg g^{-1} fw)	Raffinose (mg g^{-1} fw)	Citrate	Succinate
Genotype (G)	Moneymaker	0.47^{b}	0.42^{b}	0.23^{b}	0.34	39.52^{b}	2.70^{c}
	Negro Yeste	0.64^{a}	0.71^{a}	0.43^{a}	0.28	46.92^{a}	4.71^{b}
	Verdal	0.46^{b}	0.66^{a}	0.41^{a}	0.32	35.82^{b}	7.63^{a}
	P-Value	***	***	***	n.s.	**	***
Condition (C)	Control	0.51	0.63^{a}	0.39^{a}	0.34	47.12^{a}	4.25^{b}
	Salt Stress	0.53	0.56^{b}	0.32^{b}	0.28	34.39^{b}	5.78^{a}
	P-Value	n.s.	**	***	n.s.	***	*
Interaction	G x C	*	*	*	**	***	*

B		Fumarate	Malate	Formate	Glutamate	Formate / succinate	Glutamate / succinate
Genotype (G)	Moneymaker	1.08	69.07^{a}	7.22^{a}	93.70^{b}	2.76^{a}	36.80^{a}
	Negro Yeste	1.09	69.73^{a}	3.92^{c}	87.61^{b}	1.34^{b}	22.56^{b}
	Verdal	0.94	60.86^{b}	5.53^{b}	177.52^{a}	0.77^{b}	23.94^{b}
	P-Value	n.s.	*	***	***	***	**
Condition (C)	Control	0.99	65.86	7.76^{a}	94.58^{b}	2.24^{a}	25.44
	Salt Stress	1.08	67.25	3.35^{b}	144.64^{a}	1.01^{b}	30.09
	P-Value	n.s.	n.s.	***	***	***	n.s.
Interaction	G x C	n.s.	***	***	***	**	**

C		β-carotene	Violaxanthin	Neoxanthin	Lutein
Genotype (G)	Moneymaker	26.45^{a}	10.51^{a}	8.64^{a}	17.98^{b}
	Negro Yeste	19.63^{b}	5.88^{b}	5.56^{b}	13.33^{c}
	Verdal	28.75^{a}	8.88^{a}	8.32^{a}	22.45^{a}
	P-Value	***	***	***	***
Condition (C)	Control	25.51	9.87^{a}	7.83	17.98
	Salt Stress	24.38	6.98^{b}	7.18	17.86
	P-Value	n.s.	**	n.s.	n.s.
Interaction	G x C	*	*	*	*

D		Chlorophyll a (mg g^{-1} fw)	Chlorophyll b (mg g^{-1} fw)	Chlorophyll / carotenoid
Genotype (G)	Moneymaker	1.52^{a}	0.57^{a}	7.20^{a}
	Negro Yeste	1.32^{c}	0,44^{b}	6.66^{b}
	Verdal	1.42^{b}	0.54^{a}	7.14^{a}
	P-Value	***	***	**
Condition (C)	Control	1.43	0.50	7.21^{a}
	Salt Stress	1.41	0.53	6.79^{b}
	P-Value	n.s.	n.s.	**
Interaction	G x C	*	*	*

Effect of the genotype and salt stress condition on metabolite contents in green fruit of commercial cv. Moneymaker and traditional varieties Negro Yeste and Verdal (corresponding to results in table 2).

A		Sucrose (mg g-1 fw)	Fructose (mg g-1 fw)	Glucose (mg g-1 fw)	Mannose	UDP-Glucose	Citrate (mg g-1 fw)	Succinate
Genotype (G)	Moneymaker	0.45[b]	8.25[a,b]	10.62[a]	13.72[b]	35.16[b]	0.76[b]	90.05[a]
	Negro Yeste	0.92[a]	8.92[a]	12.04[a]	15.93[b]	40.31[b]	1.11[a]	107.83[a]
	Verdal	1.14[a]	7.26[b]	8.10[b]	22.52[a]	50.81[a]	0.71[b]	41.47[b]
	P-Value	***	*	***	**	***	***	***
Condition (C)	Control	0.30[b]	8.30	10.28	12.97[b]	40.64	0.75[b]	56.00[b]
	Salt Stress	1.37[a]	7.98	10.23	21.81[a]	43.54	0.97[a]	103.57[a]
	P-Value	***	n.s.	n.s.	***	n.s.	***	***
Interaction	G x C	**	n.s.	*	***	*	*	**

B		Fumarate	Malate (mg g-1 fw)	Formate	Glutamate (mg g-1 fw)	Formate / succinate	Glutamate / succinate
Genotype (G)	Moneymaker	4.46[a,b]	1.80[a]	2.06[a,b]	0.23[b]	0.04[a,b]	4.16[b]
	Negro Yeste	5.18[a]	1.54[b]	1.37[b]	0.26[b]	0.01[b]	2.36[b]
	Verdal	3.52[b]	0.97[c]	2.52[a]	0.40[a]	0.06[a]	9.52[a]
	P-Value	*	***	**	**	**	***
Condition (C)	Control	4.87[a]	1.46	1.78	0.20[b]	0.04	4.93
	Salt Stress	3.90[b]	1.42	2.18	0.39[a]	0.03	5.77
	P-Value	*	n.s.	n.s.	***	n.s.	n.s.
Interaction	G x C	*	*	*	*	*	***

C		Phytoene	β-carotene	Lutein	Chlorophyll a	Chlorophyll b	Chlorophyll / carotenoid
Genotype (G)	Moneymaker	2.36[c]	1.22[b]	0.88[c]	17.41[c]	5.67[c]	4.69[b]
	Negro Yeste	5.57[a]	1.70[a]	2.00[a]	37.87[a]	13.56[a]	5.28[a]
	Verdal	3.67[b]	1.72[a]	1.28[b]	31.90[b]	11.86[b]	5.38[a]
	P-Value	***	**	***	***	***	*
Condition (C)	Control	3.23[b]	1.42[b]	0.86[b]	21.79[b]	7.80[b]	5.28
	Salt Stress	4.50[a]	1.67[a]	1.92[a]	36.33[a]	12.92[a]	4.95
	P-Value	***	*	***	***	***	n.s.
Interaction	G x C	*	*	*	***	***	n.s.

Effect of the genotype and salt stress condition on metabolite contents in ripe fruit of commercial cv. Moneymaker and traditional varieties Negro Yeste and Verdal (corresponding to results in table 2).

A		Sucrose (mg g^{-1} fw)	Fructose (mg g^{-1} fw)	Glucose (mg g^{-1} fw)	Mannose	UDP-Glucose	Citrate (mg g^{-1} fw)	Succinate
Genotype (G)	Moneymaker	0.05^{b}	8.41^{b}	12.02^{a,b}	32.03^{b}	24.28^{b}	1.01^{a}	101.60^{a}
	Negro Yeste	0.18^{b}	10.36^{a}	13.28^{a}	31.59^{b}	20.36^{a,b}	1.12^{a}	95.10^{a}
	Verdal	0.46^{a}	8.88^{b}	10.56^{b}	55.43^{a}	30.56^{a}	0.59^{b}	35.00^{b}
	P-Value	***	***	***	***	*	***	**
Condition (C)	Control	0.07^{b}	8.60^{b}	10.42^{b}	36.43	25.32	0.912	84.44
	Salt Stress	0.38^{a}	9.83^{a}	13.48^{a}	42.94	24.81	0.907	69.99
	P-Value	***	***	***	n.s.	n.s.	n.s.	n.s.
Interaction	G x C	**	*	*	*	*	**	*

B		Fumarate	Malate (mg g^{-1} fw)	Formate	Glutamate (mg g^{-1} fw)	Formate / succinate	Glutamate / succinate
Genotype (G)	Moneymaker	3.18^{a}	1.09^{b}	1.50^{b}	1.30^{b}	0.02^{b}	13.48^{b}
	Negro Yeste	1.42^{b}	1.47^{a}	1.31^{b}	1.75^{a,b}	0.02^{b}	22.45^{b}
	Verdal	0.70^{b}	0.48^{c}	16.90^{a}	2.25^{a}	0.61^{a}	72.67^{a}
	P-Value	***	***	***	**	**	***
Condition (C)	Control	2.10	1.16^{a}	3.84^{b}	1.69	0.11	30.30
	Salt Stress	1.43	0.87^{b}	9.30^{a}	1.85	0.32	42.11
	P-Value	n.s.	**	***	n.s.	n.s.	n.s.
Interaction	G x C	*	*	***	*	*	*

C		Phytoene	β-carotene	Lycopene	Lutein	Chlorophyll a	Chlorophyll b
Genotype (G)	Moneymaker	6.17^{a}	2.94^{b}	64.48^{b}	0.30^{b}	ND^{c}	ND^{c}
	Negro Yeste	2.54^{b}	6.12^{a}	120.69^{a}	1.74^{a}	21.72^{a}	6.17^{a}
	Verdal	1.34^{c}	1.04^{c}	ND^{c}	0.50^{b}	15.03^{b}	5.93^{a}
	P-Value	***	***	***	***	***	***
Condition (C)	Control	2.78^{b}	3.46	52.44^{b}	0.65^{b}	9.34^{b}	3.10^{b}
	Salt Stress	3.92^{a}	3.27	71.02^{a}	1.04^{a}	15.16^{a}	4.96^{a}
	P-Value	***	n.s.	***	***	***	***
Interaction	G x C	***	*	**	*	**	**

D		Chlorophyll / carotenoid
Genotype (G)	Moneymaker	0.00^{b}
	Negro Yeste	0.20^{b}
	Verdal	4.26^{a}
	P-Value	***
Condition (C)	Control	1.74^{a}
	Salt Stress	1.24^{b}
	P-Value	**
Interaction	G x C	***

Metabolites contents are presented as µg g^{-1} fresh weigh (fw) unless otherwise stated in the table. Different letters in each principal factor indicate statistically significant differences at $P < 0.05$ (Tukey's test). *, ** and *** indicates significant differences at $P \leq 0.05$, $P \leq 0.01$ and $P \leq 0.001$ respectively for each principal factor and the interaction. n.s. = no significance.
