# Supplementary material for: Recovering Tomato Landraces to Simultaneously Improve Fruit Yield and Nutritional Quality Against Salt Stress
Source: Front Plant Sci. 2018 Nov 30;9:1778. doi: 10.3389/fpls.2018.01778 (PMC6284034; doi:10.3389/fpls.2018.01778)
Supplement: Supplementary file 3 [file Image_1.pdf]

A

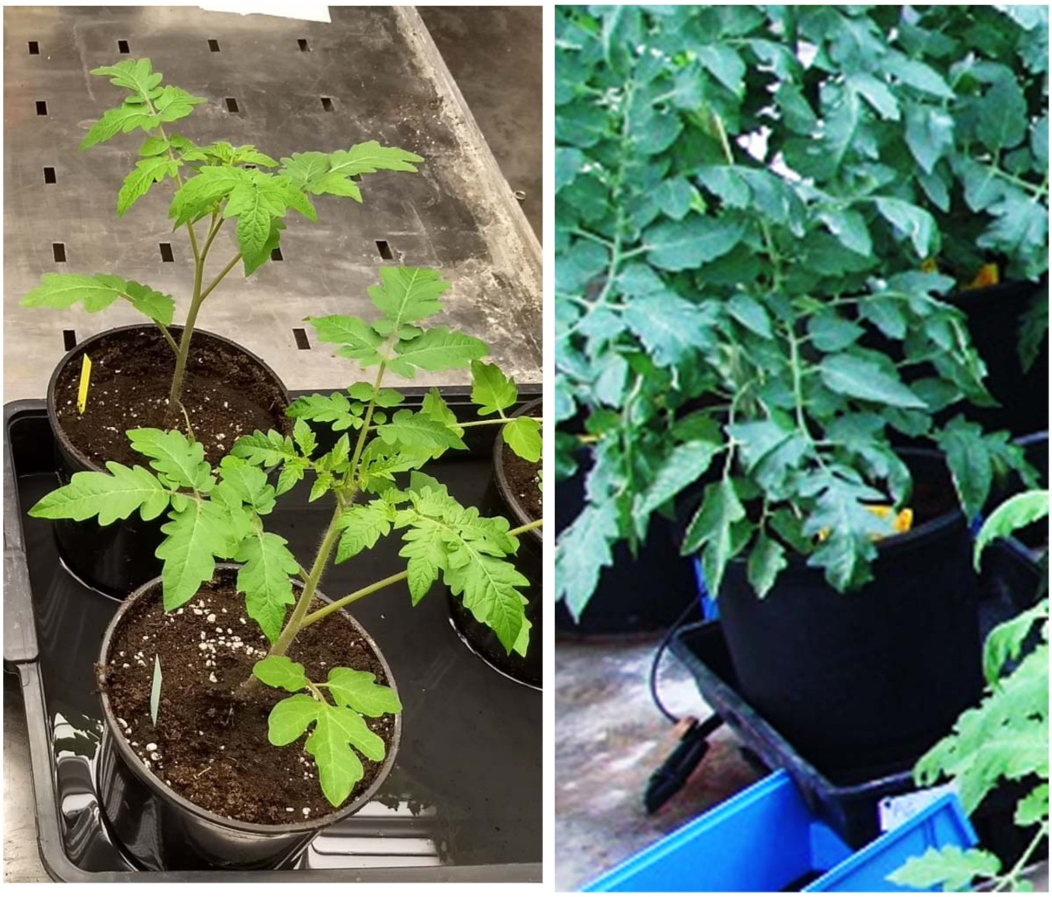

B

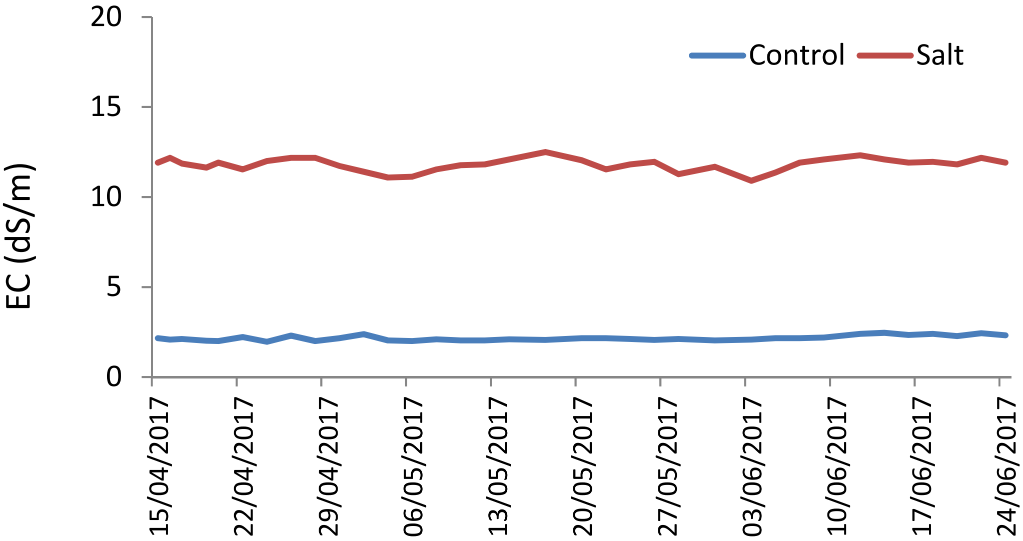

Supplementary Figure 1. (**A**) Images of the plant culture: pots used, coco peat substrate and trays (blue colour) where the lixiviate was collected. The pictures correspond to plants just after transplanting to pots (left) and at the end of the salt stress assay (right). (**B**) EC values of the lixiviates in control and saline (100 mM NaCl) growth conditions for the whole duration of the assay (70 days).
